# Supplementary material for: Distributions of recorded pain in mental health records: a natural language processing based study
Source: BMJ Open. 2024 Apr 19;14(4):e079923. doi: 10.1136/bmjopen-2023-079923 (PMC11033644; doi:10.1136/bmjopen-2023-079923)
Supplement: Author's manuscript [file bmjopen-2023-079923.draft_revisions.pdf]

BMJ Open is committed to open peer review. As part of this commitment we make the peer review history of every article we publish publicly available.

When an article is published we post the peer reviewers' comments and the authors' responses online. We also post the versions of the paper that were used during peer review. These are the versions that the peer review comments apply to.

The versions of the paper that follow are the versions that were submitted during the peer review process. They are not the versions of record or the final published versions. They should not be cited or distributed as the published version of this manuscript.

BMJ Open is an open access journal and the full, final, typeset and author-corrected version of record of the manuscript is available on our site with no access controls, subscription charges or pay-per-view fees (http://bmjopen.bmj.com).

If you have any questions on BMJ Open's open peer review process please email info.bmjopen@bmj.com

BMJ Open

Distributions of Recorded Pain in Mental Health Records: A Natural Language Processing Based Study

Journal:	*BMJ Open*
Manuscript ID	bmjopen-2023-079923
Article Type:	Original research
Date Submitted by the Author:	18-Sep-2023
Complete List of Authors:	Chaturvedi, Jaya; King's College London, Department of Biostatistics and Health Informatics<br>Stewart, Robert; King's College London, Institute of Psychiatry<br>Ashworth, Mark; King's College London, School of Population Health & Environmental Sciences<br>Roberts, Angus; King's College London, Institute of Psychiatry, Psychology and Neuroscience
Keywords:	Natural Language Processing, Electronic Health Records, EPIDEMIOLOGY, MENTAL HEALTH, Chronic Pain

SCHOLARONE™
Manuscripts

BMJ

I, the Submitting Author has the right to grant and does grant on behalf of all authors of the Work (as defined in the below author licence), an exclusive licence and/or a non-exclusive licence for contributions from authors who are: i) UK Crown employees; ii) where BMJ has agreed a CC-BY licence shall apply, and/or iii) in accordance with the terms applicable for US Federal Government officers or employees acting as part of their official duties; on a worldwide, perpetual, irrevocable, royalty-free basis to BMJ Publishing Group Ltd ("BMJ") its licensees and where the relevant Journal is co-owned by BMJ to the co-owners of the Journal, to publish the Work in this journal and any other BMJ products and to exploit all rights, as set out in our licence.

The Submitting Author accepts and understands that any supply made under these terms is made by BMJ to the Submitting Author unless you are acting as an employee on behalf of your employer or a postgraduate student of an affiliated institution which is paying any applicable article publishing charge ("APC") for Open Access articles. Where the Submitting Author wishes to make the Work available on an Open Access basis (and intends to pay the relevant APC), the terms of reuse of such Open Access shall be governed by a Creative Commons licence – details of these licences and which Creative Commons licence will apply to this Work are set out in our licence referred to above.

Other than as permitted in any relevant BMJ Author's Self Archiving Policies, I confirm this Work has not been accepted for publication elsewhere, is not being considered for publication elsewhere and does not duplicate material already published. I confirm all authors consent to publication of this Work and authorise the granting of this licence.

For peer review only

Distributions of Recorded Pain in Mental Health Records: A Natural Language Processing Based Study

Jaya Chaturvedi[1*], Robert Stewart[1,2], Mark Ashworth[1], Angus Roberts[1]
[1]Institute of Psychiatry, Psychology and Neurosciences, King's College London
[2]South London and Maudsley NHS Foundation Trust
*Corresponding author: jaya.1.chaturvedi@kcl.ac.uk

For peer review only

Abstract

Objective

The objective of this study is to determine demographic and diagnostic distributions of physical pain recorded in the clinical notes of a mental health electronic health records database by utilising natural language processing and to examine the level of overlap in recorded physical pain between primary and secondary care.

Design, Setting and Participants

The data were extracted from an anonymised version of the electronic health records from a large mental community and secondary healthcare provider serving a catchment of 1.3M residents in south London. These included patients under active referral and aged 18+ at the index date of July 1, 2018, and had at least one clinical document (>=30 characters) associated with their record between July 1, 2017 and July 1, 2019. This cohort was compared to linked primary care records from one of the four catchment boroughs.

Outcome

The primary outcome of interest was the presence or absence of recorded physical pain within the clinical notes of the patients. This does not include mental, psychological or metaphorical pain.

Results

A total of 27,211 patients were retrieved based on the extraction criteria. Of these, 52% (14,202) had narrative text containing relevant mentions of physical pain. Patients who were older (OR 1.17, 95% CI 1.15-1.19), female (OR 1.42, 95% CI 1.35-1.49), of Asian (OR 1.30, 95% CI 1.16-1.45) or Black (OR 1.49, 95% CI 1.40-1.59) ethnicities, and living in deprived neighbourhoods (OR 1.64, 95% CI 1.55-1.73) showed higher odds of recorded pain. Patients with an SMI diagnosis were found to be less likely to report pain (OR 0.43, 95% CI 0.41-0.46, $p<0.001$). When comparing the overlap between primary and secondary care, 17% of the CRIS cohort also had records within LDN, and 31% of these had recorded pain in both records.

Conclusion

The findings of this study show the sociodemographic and diagnostic differences in recorded pain, and have significant implications for the assessment and management of physical pain in patients with mental health disorders.

Keywords: Natural Language Processing, Pain, Mental Health, Electronic Health Records

Word count: 3,780

Strengths and Limitations of this study

- This study utilises natural language processing on clinical notes to access a large sample with information about pain.
- This is the first cross-sectional study to summarise and describe the distribution of recorded pain within the clinical notes of mental health records.
- The recorded mentions of pain within clinical notes clearly depend on the patient sharing and the clinician recording their experiences.
- The findings are not generalisable to the general population since this study only looks at patients receiving mental healthcare within a specific geographic catchment.

Introduction

Background Rationale

Pain and its relationship with mental health are important research topics. Pain has imposed a significant burden on society in terms of medical care costs as well as lost productivity [1]. Pain is multifaceted, with physical, psychological, social, and biological causes and consequences [2]. Mental health disorders also present a considerable and complex public health problem, being a leading cause of disability and accounting for 28% of the national disease burden in the UK [3]. Electronic health records (EHRs) for mental health are a significant source of information for studying the intersection between pain and mental health within those who receive specialist service input. EHRs open the possibility of investigating how pain is recorded and its impact on clinical outcomes.

Severe mental illnesses (SMIs) include diagnoses of schizophrenia spectrum disorder, bipolar disorder, or severe major depressive disorder [4], where functional and occupational activities are severely impaired due to associated debilitating psychological problems [5]. While several studies have looked at the relationship between pain and schizophrenia and bipolar disorders [6–9] and at other mental illnesses such as depression [1,10–13], the complex and potentially bidirectional nature of this relationship requires further understanding. Analysis of secondary data sources, such as EHR databases, might help by providing a fuller picture of the recorded clinical presentation of this group of patients; however, a prerequisite is that pain is adequately represented in derived data.

For peer review only

Demographic features such as age, gender and ethnicity can influence pain perception and experiences. Pain affects twice as many persons over the age of 60 as it does younger individuals [14]. While pain is not a natural feature of the ageing process, many health conditions causing pain become more common with increasing age. Nonetheless, older patients often believe pain to be a normal aspect of ageing and might be hesitant when reporting it [14]. There have also been variations found in the reported perception of pain by female and male patients, with female patients reporting experiencing more pain than males [15,16]. Research has also shown disparities in pain perception across different ethnicities, with individuals of Black (African) ethnicity reporting greater pain than White counterparts [17].

Socioeconomic status (SES) plays a role in health and overall well-being, with deprivation associated with unfavourable health outcomes and increased mortality rates [18]. Patients with SMI already experience higher mortality rates than the general population, and this discrepancy is exacerbated by socioeconomic deprivation, primarily due to unequal access to good quality physical healthcare services [19–22]. Furthermore, patients with SMI continue to experience a decline in their SES over time, compounding its impact [23].

Most patient information is recorded in unstructured clinical narratives within EHR databases [24], and pain is likely to be no different, with few, if any, structured checklists ascertaining its presence in routine clinical care. Natural language processing (NLP), a computational approach to understanding and analysing human language, is therefore potentially useful for extracting such pain information. NLP has been applied extensively to EHR data, including studies of SMI, such as antipsychotic polypharmacy in mental health care [25], multimorbidity in individuals with schizophrenia and bipolar disorders [26], and extracting symptoms of SMI [27].

In addition to secondary care data, it is also useful to consider the recording of pain in primary care data. Within the UK, primary care is generally the first point of contact for patients [28]. Exploring the overlap of recorded pain between primary and secondary care could, therefore, provide a more comprehensive view of the patient's pain experiences, and any discrepancies could highlight gaps in care and communication.

Objectives

The objective of this study is to describe the distributions of recorded pain amongst mental health service users according to demographic factors such as age, gender and ethnicity, as well as neighbourhood deprivation levels and mental health diagnoses. This was achieved by examining recorded pain through the means of an NLP application within the clinical text of a mental health EHR database, and further evaluating this by measuring the overlap between pain recorded in secondary and primary health care, enabled through data linkage between the two.

For peer review only

Methods

Reporting

We use the RECORD [29] guidelines and checklist, an extension of the STROBE [30] guidelines, for reporting the results of this study.

Setting

Data on recorded pain were obtained from the clinical text of a mental health EHR database, the Clinical Record Interactive Search (CRIS) resource. This contains a de-identified version of EHR data from The South London and Maudsley NHS Foundation Trust (SLaM), one of Europe's largest mental healthcare organisations [31], which serves a geographic catchment of around 1.3 million residents in four south London boroughs (Croydon, Lambeth, Lewisham, Southwark). CRIS contains about 30 million free text documents, averaging 90 documents per patient [24].

Data were also obtained from a primary care database called Lambeth DataNet (LDN) [32], which accesses all GP records from general practices based in the London borough of Lambeth. Data linkages (at the patient level) are already in place between CRIS and LDN [33].

Ethical Approval

CRIS (as well as its associated linkages) has received ethical approval as a data resource for secondary analysis from the Oxford C Research Ethics Committee (reference 23/SC/0257). A patient-led oversight committee (detailed in [34]) reviews and approves research projects that use the CRIS database. For service users, an opt-out system is in place and is advertised in all promotional materials and campaigns. Only authorised individuals can access this data from within a secure firewall. The CRIS project approval references for this work are 21-021 and 23-003.

LDN approval was obtained as part of an existing CRIS project (project number 23-124) which included access to linked data from LDN (Caldicott Guardian approval, 15/9/21). This CRIS-LDN project aimed to examine the profile of patients with mental illnesses and chronic/persistent pain and compare them to controls from LDN who had chronic/persistent pain only.

Patient and Public Involvement

Patient and public involvement (PPI) in research is an active collaboration between researchers and members of the public, where the latter actively participate in contributing to the research [35]. A PPI group with lived experiences of SMI and chronic pain were consulted as part of this research. The nature of the data available was described to the group, and they were asked about their priorities regarding what research questions they would like answered. In response to this, the group was unanimously interested in further study of the differences in pain experiences based on demographics and diagnoses, and this was the main motivation for the objective of the study described here.

For peer review only - http://bmjopen.bmj.com/site/about/guidelines.xhtml

Participants

A cohort of patients was extracted from the CRIS database comprising those who were active (i.e., under an accepted referral) and aged 18+ on the index date of July 1, 2018, and whose record contained at least one document (>= 30 characters) within a window of July 1, 2017 to July 1, 2019.

LDN extraction followed similar criteria for patients who were active on the index date, aged 18+, and contained pain diagnoses or medications from July 1, 2017 to July 1, 2019. Free-text information is unavailable within LDN, so no document criteria were required.

Variables

Demographics

Age, gender, and ethnicity variables were extracted from structured tables within the CRIS database. Individuals with missing ethnicity values were retained as a separate category (Not stated/known).

Diagnosis

The primary diagnosis recorded closest to the index date of July 1, 2018, was extracted from the structured tables within the CRIS database. These are coded using ICD-10 [36]. The diagnosis codes were categorised as SMI (severe mental illnesses) and non-SMI, where SMI includes ICD-10 codes of F20-29 and F30-33.

Deprivation

The Index of Multiple Deprivation (IMD) decile measures from 2019 [37] were extracted for information on neighbourhood deprivation for each patient, based on their address at the time of the index date aggregated by Lower Super Output Area (LSOA) - a standard national administrative unit containing an average 1500 residents. National Census data are used to calculate IMD scores for each LSOA. A lower IMD decile indicates higher deprivation levels. Individuals with missing IMD scores were retained in a separate category.

Recorded Pain

Pain-related keywords generated from a lexicon of pain terms [38] were used to identify patients in the cohort who had mentions of physical pain recorded in their clinical notes within the predetermined window. An NLP application was used on the documents of these patients. The application classified sentences within the document as relevant or not, where relevant refers to a mention of physical pain affecting the patient, and not relevant refers to no or negated mentions, hypothetical mentions, and metaphorical mentions of pain. Only relevant mentions were used in the results reported here. The application has been described in detail in [39].

As with all other UK research based on access to anonymised primary care records, LDN does not allow access to any free text clinical notes. For this reason, pain information can only

For peer review only

be extracted from the structured fields of the records. Read codes [40] were used to identify patients who had a pain diagnosis or were on any pain medications and treatments:

1. Pain medications code list - developed as part of a project described in [41], which focused on analgesics (obtained from dm+d (a dictionary of medicines and devices [42]) used in the treatment of 35 long-term conditions. These 35 conditions were obtained from [43], a cross-sectional study on multimorbidities in patients registered with 314 medical practices in Scotland as of March 2007.
2. Pain diagnosis and treatments code list - developed as part of a collaboration project with Outcomes Based Healthcare (OBH), an organisation that provides a platform for the study of population health outcomes [44], with the research described in [45].

While these codes were developed for chronic pain, they are generic enough to be used for this research. These code lists are available on GitHub[1].

Anatomy Related to Recorded Pain

Another NLP application was developed for identifying anatomy mentioned in relation to pain. This was a classifier that generated a binary output - “mentioned” or “not mentioned”. This application was run on sentences labelled as relevant by the pain application. Once the sentences that contained mentions of body parts were identified, they were run through MedCATTrainer [46], which used named entity recognition (NER), a type of NLP task to label entities within the text to identify the specific body parts mentioned within the text. The purpose of using MedCATTrainer was that it linked the identified body parts to unique identification numbers (SCTID) from SNOMED CT, a terminology of clinical terms. These SCTIDs were used to aggregate the mentioned body parts, for ease of analysis. For example, foot, calf, and knee mentions would be aggregated under “lower limb”.

Overlap between CRIS and LDN

To examine the overlap across primary (LDN) and secondary (CRIS) care, the patient IDs from the CRIS cohort (N=27,211) were searched for matching records within the LDN database over the same window of July 1, 2017 to July 1, 2019. Variables were generated indicating the presence of the patients within LDN, along with variables indicating the presence of any codes for pain medication, diagnosis or treatment based on the predefined lists described above. This allowed the identification of patients with documented pain experiences in both their mental health and primary care records for the aligned time period. The cross-referencing process enabled the comparison of recorded pain between the two systems at the patient level.

Descriptive Statistics

All analysis was conducted using STATA v15.1 and the Python programming language (version 3.10.0).

Descriptive statistics were obtained for demographic, deprivation and diagnosis features and compared between the two groups - patients who had recorded pain (class 1) and those who

[1] https://github.com/jayachaturvedi/pain_in_mental_health

For peer review only

did not (class 0) - within their clinical notes. Chi-squared tests and logistic regression were conducted between the two classes to obtain adjusted odds ratios. Frequencies of body parts affected by pain and the overlap of recorded pain experiences between CRIS and LDN were also reported.

Results

Data Extraction

Based on the extraction criteria, 27,211 patients were represented. Amongst these patients, 18,188 had pain keywords mentioned within their documents. These documents were run through the NLP application to label them as relevant to pain (class 1) or not (class 0), resulting in 14,202 patients who had relevant mentions of pain within their clinical notes (Figure 1).

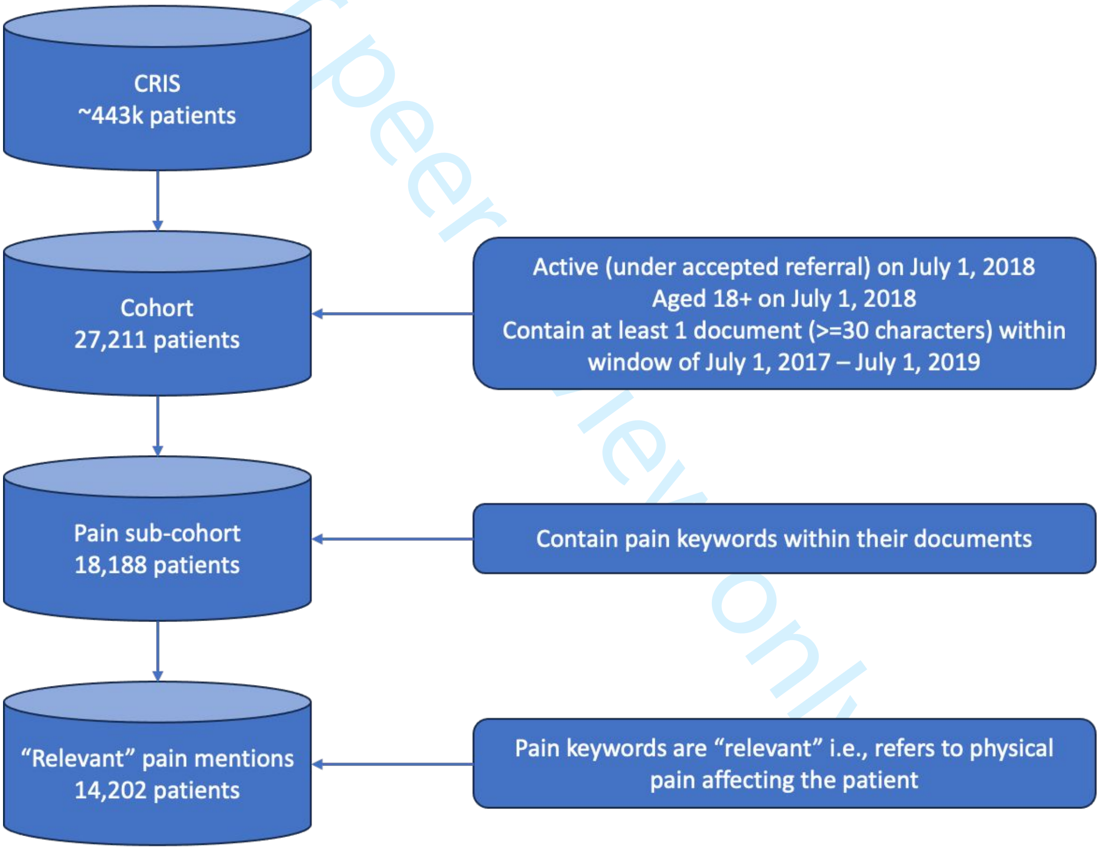

Figure 1. Data Extraction

Cohort Characteristics

Amongst the cohort of 27,211 patients, the mean age of the cohort was 44 (Inter-quartile range 29-55, SD 17.5), with 50.3% female and 48.2% of White ethnicity. The majority of the cohort (72.2%) lived in more deprived areas (IMD score <=5), and 67.0% received a non-SMI diagnosis. 66.8% of the patients (18,188 patients and 174,167 mentions within documents) contained pain keywords within their documents, and 52.1% of the cohort (14,202 patients) contained relevant mentions of pain in their documents.

Pain Mentions

Records of 52.1% of the patients within the cohort contained relevant mentions of pain. Differences between the patients who showed recorded pain (class 1) in their clinical notes and those who didn't (class 0) are shown in Table 1. Class 0 includes patients who did not have any pain mentions in their documents, as well as patients whose pain mentions were classified as not relevant. Patients within class 1 had an average of 10 pain mentions within their documents.

**Characteristic**	**n**	**Class 0 (no recorded pain)**	**Class 1 (recorded pain)**
N (%)	27,211	13,009 (47.9)	14,202 (52.1)
Mean Age (IQR)	44 (29–55)	41 (27–52)	46 (32–56)
Gender (N, %)			
Male	13,471	7,037 (54.1)	6,434 (45.3)
Female	13,709	5,953 (45.7)	7,756 (54.6)
Not known	31	19 (0.2)	12 (0.1)
Ethnicity (N, %)			
White	13,139	6,014 (46.2)	7,125 (50.1)
Black	5,866	2,115 (16.2)	3,751 (26.4)

For peer review only

Not stated/known	4,708	3,418 (26.2)	1,290 (9.0)
Asian	1,506	592 (4.5)	914 (6.4)
Other	1,197	512 (3.9)	685 (4.8)
Mixed	795	358 (2.7)	437 (3.0)
Index of Multiple Deprivation (N, %) Decile 2019			
<= 5 (more deprived)	19,660	8,847 (68.0)	10,813 (76.1)
> 5 (less deprived)	6,686	3,836 (29.4)	2,850 (20.0)
Not known	865	326 (2.5)	539 (3.9)
Primary Diagnosis: SMI vs Non-SMI (ICD-9 code) (N, %)			
SMI	8,962	3,059 (23.5)	5,903 (41.5)
Non-SMI	18,249	9,950 (76.5)	8,299 (58.5)

Table 1. Distributions between the two classes - class 0 (no recorded pain) and class 1 (recorded pain)

Demographic variations emerged between those with/without recorded pain in the cohort, as shown in Table 1. The mean age was higher in patients with recorded pain at 46 (SD=17) compared to 41 (SD=17) for the remainder. Patients with recorded pain were more likely to be female and had a higher representation across all ethnic minorities. Additionally, patients with documented pain experiences were more likely to live in higher deprivation neighbourhoods.

For peer review only

Table 2 presents demographic, deprivation and diagnostic associations with recorded pain obtained through logistic regressions (unadjusted and adjusted for different factors as detailed below).

	Logistic Regression models				
	Unadjusted	Mutually adjusted			
		Model 1	Model 2	Model 3	Model 4
**Age** (per 10 years)	1.17 [1.15, 1.19] *	1.12 [1.11, 1.14] *	1.12 [1.11, 1.14] *	1.11 [1.10, 1.13] *	-
**Gender**					
Male	1 (reference)	1 (reference)	1 (reference)	1 (reference)	-
Female	1.42 [1.35, 1.49] *	1.42 [1.35, 1.49] *	1.43 [1.36, 1.50] *	1.47 [1.40, 1.55] *	-
Not known	0.69 [0.33, 1.42]	1.08 [0.50, 2.33]	1.06 [0.49, 2.30]	1.10 [0.51, 2.38]	-
**Ethnicity**					
White	1 (reference)	1 (reference)	1 (reference)	1 (reference)	1 (reference)
Asian	1.30 [1.16, 1.45] *	1.36 [1.22, 1.52] *	1.34 [1.19, 1.49] *	1.21 [1.08, 1.36] *	1.29 [1.15, 1.44] *
Black	1.49 [1.40, 1.59] *	1.58 [1.48, 1.69] *	1.50 [1.40, 1.60] *	1.25 [1.17, 1.34]	1.42 [1.33, 1.52] *
Other	1.12 [1.00, 1.27]	1.20 [1.06, 1.36]	1.17 [1.03, 1.32]	1.10 [0.97, 1.24]	1.08 [0.96, 1.33]
Mixed	1.03 [0.89, 1.18]	1.15 [0.99, 1.33]	1.12 [0.96, 1.30]	1.06 [0.91, 1.23]	1.01 [0.87, 1.17]
Not known	0.31 [0.29, 0.34] *	0.36 [0.34, 0.39] *	0.37 [0.34, 0.40] *	0.40 [0.37, 0.44] *	0.32 [0.30, 0.35] *
**Index of Multiple Deprivation**					
National Decile <=5	1.64 [1.55, 1.73] *	-	1.43 [1.35, 1.51] *	1.37 [1.29, 1.45] *	1.41 [1.33, 1.50] *
**Diagnosis**					
SMI	0.43 [0.41, 0.46] *	-	-	0.56 [0.53, 0.59] *	-

Table 2. Logistic Regression findings for variables reflecting differences in class 0 (no recorded pain) and class 1 (recorded pain) (N = 27,211)
Values are given as odds ratio (95% CI), and * indicates significance at $p < 0.001$
Model 1 contained the demographic variables only [age, gender and ethnicity].
Model 2 contained the variables from Model 1, plus the variable for deprivation (IMD Decile].

Model 3 contained the variables from Model 2 plus the diagnosis variable.
Model 4 contains the ethnicity and deprivation variables alone.

Unadjusted odds ratios revealed patients with documented pain experiences were more likely to be older (OR 1.17, 95% CI 1.15-1.19, p<0.001), female (OR 1.42, 95% CI 1.35-1.49, p<0.001), of Asian (OR 1.30 in relation to a White reference group, 95% CI 1.16-1.45, p<0.001) or Black (OR 1.49, 95% CI 1.40-1.59, p<0.001) ethnicities, and living in deprived neighbourhoods (OR 1.64, 95% CI 1.55-1.73, p<0.001) when compared to the remainder of the sample. In a model containing all demographic variables (Model 1), the odds ratios were strengthened for all ethnic minority groups. Additional adjustment for neighbourhood deprivation (Model 2) resulted in a further strengthening of the odds ratio for females. In the model also adjusted for diagnoses (Model 3), odds ratios became stronger for females. Patients with SMI had lower odds of documented pain (OR 0.43, 95% CI 0.41-0.46, p<0.001) than non-SMI patients, with the odds ratio slightly weakening when adjusted for demographics, deprivation and diagnosis (Model 3). A supplementary model (Model 4) including both ethnicity and deprivation as covariates showed independent increased odds for Asian and Black patients and those in more deprived neighbourhoods.

Anatomy Distributions

Additional descriptive data were generated on the nature of the pain reported. Amongst the 14,202 patients with any recorded pain, there were 174,167 mentions of pain within the documents. Of these, 7,555 (53%) patients included 40,418 mentions of the anatomy associated with the pain. Of these 53%, each patient had an average of 5 body parts mentioned in the context of pain. The most common body part affected by pain, as per the recorded mentions, was lower limbs, which accounted for 20% of all mentions where anatomy could be ascertained (Table 3).

Body Part	Mentions	Frequency (mention-level)
Lower limbs	Feet, ankle, leg, knee, calf, thigh, toes	20%
Upper body, excluding back	Chest, side of chest, upper body, torso	19%
Upper limbs	Hand, wrist, arm, elbow, thumb, shoulder	17%
Stomach/abdomen region	Stomach, abdomen, groin, bladder, prostate	16%
Head and neck	Head, tooth, face, mouth, tongue, eye, ear, neck	15%

For peer review

Non-specific site	Entire body, skin, muscle, joint	8%
Back	Back, lower back	5%

Table 3. Body parts affected (at mention level)

Overlap with Primary Care

When comparing secondary care CRIS records with those of primary care from LDN, among the 27,211 patients of the CRIS cohort, 4,822 patients (17%) also had records in LDN. Amongst these patients who had records in both CRIS and LDN, 1,507 (31%) patients were identified as having some recorded instance of pain in both their records, while 687 (14%) patients showed recorded pain only in LDN (primary care). Among the 27,211 patients within CRIS, 12,695 (46%) had recorded pain only within CRIS (mental health care), as seen in Figure 2.

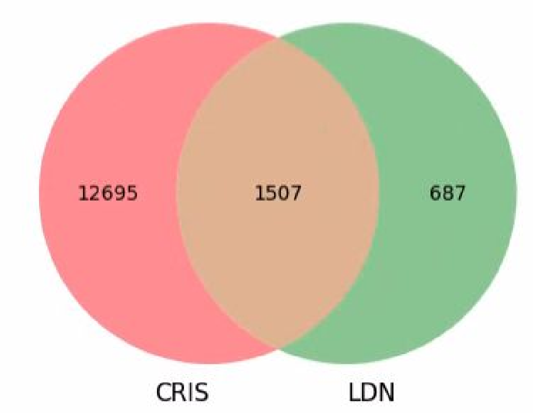

Figure 2. Overlap of recorded pain in CRIS and LDN

Discussion

This study investigated the differences observed in recorded pain mentions within the clinical notes of mental health records. The results reflect current literature findings that pain is a common issue among patients with mental health disorders. In a cohort of 27,211 patients, 18,188 (67%) patients contained pain-related keywords in their text, and 14,202 (52%) patients had relevant pain mentions, i.e., the mention indicated physical pain affecting the patient in question. Disparities in recorded pain mentions were found across genders, with females being over-represented. This is consistent with other research that indicates gender disparities in pain experiences [16,48,49]. Furthermore, while patients with known ethnicities were mostly over-represented in the cohort of relevant pain mentions (in relation to those with unknown ethnicity), most noticeable were the Black, Asian and other ethnic groups. This aligns with research around the undertreatment of patients within certain ethnic minority groups [50] and highlights the need for a comprehensive exploration of pain experiences across diverse populations. Moreover, the study's findings are also consistent with studies that indicate the

impact of deprivation on health outcomes [18], as people living in more deprived areas (IMD decile <= 5) were more frequently recorded with pain.

When comparing the overlap of patients between primary and secondary care, it was found that 17% of the patients within the CRIS cohort also had records within LDN. Amongst these patients, 31% had recorded pain instances in both records. While this overlap between primary and secondary care seems low, it is important to bear in mind that Lambeth only represents 22% of the catchment covered by CRIS [47]. Patients present in CRIS but not in LDN could include patients who have recorded instances of pain within the free-text clinical notes in LDN and might have been missed in this study since we do not have access to this text. Furthermore, this study did not differentiate between acute and chronic pain mentions and focused on extracting mentions of physical pain of any duration. As a result, the higher occurrence of pain mentioned within CRIS can be partially attributed to the documentation of such acute or short-lived pain episodes. Conversely, the GP records within LDN likely focus on recording persistent and chronic pain experiences. This disparity in recording pain should be considered when interpreting the findings of this study. Looking specifically at chronic pain instances within the CRIS notes may improve the comparability. However, the temporal information required to determine pain chronicity from clinical notes is a particular challenge and can be difficult to extract reliably. Future work can attempt to differentiate acute and chronic pain through temporal or contextual information, which could provide richer insight. However, the current broad inclusion of pain provides wider coverage for this initial exploration of pain mentioned within clinical notes.

A strength of this study is the size of the data set available and the access to information about pain from the clinical text. To the best of our knowledge, this is potentially the first cross-sectional study to summarise and describe the distribution of recorded pain derived from routine mental health records. While the cohort data extraction did not apply any filters on demographics, aiming for broad representativeness, other systemic biases related to access to healthcare resources may still exist. Factors like deprivation level and ethnicity can influence the utilisation of services and, therefore, documentation within health records, often stemming from perceived barriers to access. However, by not restricting cohort selection on demographic factors, this study intended to capture a diverse patient population receiving care across the South London boroughs.

A limitation of this study is that the recorded mentions of pain within clinical notes depend on the clinician recording them. The actual occurrences of pain experiences could remain unaccounted for if they weren’t recorded by the clinicians or were not shared with the clinicians, especially for patients with severe mental illnesses who might be completely or partially nonverbal. While the NLP application achieved good performance metrics during its development and evaluation, it is not impervious to imperfections. Instances of pain experiences might have been overlooked if they were not included as examples during the training of the application.

The scope of this study is limited to the examination of mental health records from an EHR database in South London. Given the absence of a comparative cohort of patients experiencing pain without any mental health disorders, the findings of this study are not generalisable to the overall population. However, they might be relevant and generalisable to some extent to other populations of patients with mental health disorders. It is essential to

For peer review only

acknowledge the potential influence of gender and ethnicity on the reporting of pain experiences, particularly if females and minority ethnicities (due to language barriers or other reasons) are less likely to self-report their pain experiences [50,52,53]. Since the focus of this study has been on a mental health EHR database, the clinical care within this setting is focused on mental health issues reported by the patients. Consequently, as much importance might not be given to the investigation and reporting of physical health conditions such as pain.

This study cannot determine a cause-and-effect relationship or directionality between pain and mental illnesses. Despite this, the study has highlighted existing disparities in recorded pain experiences and brings to attention the need for further research to better understand and address them at the point of care.

Conclusion

The outcomes of this study have significant implications for the assessment and management of pain amongst patients with mental health disorders and highlight the importance of utilising NLP methods on EHR databases for research purposes. Notably, these findings reiterate the recommendations set forth by Mental Health America [54], advocating the need for proactive initiation of conversations around mental health and pain with patients. Relying solely on patients to self-report symptoms could potentially lead to worse outcomes, especially since the stigma surrounding pain and mental health conditions may prevent patients from seeking the necessary treatment. Thus, early and proactive interventions could go a long way towards improved long-term outcomes. Unfortunately, there still exists a perceived lack of credibility and empathy towards patients living with pain [55], particularly when compounded by co-existent mental illnesses. This was one of the main points shared by the PPI group consulted as part of this study. More research in this area can help towards these issues and provide safer and equitable access to good-quality pain management.

While these findings represent a step forward, they are only one side of the story. Combining these findings with patient-reported insights could offer a more comprehensive understanding of pain experiences within this cohort. However, achieving this is a challenging task due to the lack of such data and the inability to link patient-reported experiences to their health records. Further research is needed to better understand the relationship between pain and mental health and to develop more effective interventions to manage pain in this population.

Data Availability Statement

Data are owned by a third party, Maudsley Biomedical Research Centre (BRC) Clinical Records Interactive Search (CRIS) tool, which provides access to anonymised data derived from SLaM electronic medical records. These data can be accessed by permitted individuals from within a secure firewall (i.e. the data cannot be sent elsewhere) in the same manner as the authors. For more information, please contact cris.administrator@slam.nhs.uk. Any STATA and Python code used in this project will be available on GitHub[2].

[2] https://github.com/jayachaturvedi/pain_in_mental_health

For peer review only

Author Contributions

The idea was conceived by JC, AR, and RS. JC conducted the analyses and drafted the manuscript. MA provided insights on LDN data. AR and RS provided guidance in the design and interpretation of results. All authors commented on drafts of the manuscript and approved the final version.

Funding and Acknowledgements

AR is funded by Health Data Research UK, an initiative funded by UK Research and Innovation, Department of Health and Social Care (England) and the devolved administrations, and leading medical research charities. RS is part-funded by i) the National Institute for Health Research (NIHR) Biomedical Research Centre at South London and Maudsley NHS Foundation Trust and King's College London; ii) the National Institute for Health Research (NIHR) Applied Research Collaboration South London (NIHR ARC South London) at King's College Hospital NHS Foundation Trust; iii) the DATAMIND HDR UK Mental Health Data Hub (MRC grant MR/W014386); iv) the UK Prevention Research Partnership (Violence, Health and Society; MR-VO49879/1), an initiative funded by UK Research and Innovation Councils, the Department of Health and Social Care (England) and the UK devolved administrations, and leading health research charities. JC is supported by the KCL-funded Centre for Doctoral Training (CDT) in Data-Driven Health. The funders were not involved in the study design, collection, analysis, interpretation of data, the writing of this article or the decision to submit it for publication. RS declared research support received in the last 36 months from Janssen, GSK and Takeda. All other authors declare no other competing interests.

This paper represents independent research part-funded by the National Institute for Health Research (NIHR) Biomedical Research Centre at South London and Maudsley NHS Foundation Trust and King's College London. The views expressed are those of the authors and not necessarily those of the NHS, the NIHR or the Department of Health and Social Care. The funders had no role in study design, data collection and analysis, decision to publish, or preparation of the manuscript.

This work uses data provided by patients and collected by the NHS as part of their care and support. An application for access to the Clinical Record Interactive Search (CRIS) database for this project was submitted and approved by the CRIS Oversight Committee. The authors would like to acknowledge Dr Ruimin Ma for her help in obtaining the LDN codes.

Bibliography

1 Rayner L, Hotopf M, Petkova H, *et al.* Depression in patients with chronic pain attending a specialised pain treatment centre: prevalence and impact on health care costs. *Pain* 2016;**157**:1472–9. doi:10.1097/j.pain.0000000000000542

2 Merlin JS, Zinski A, Norton WE, *et al.* A conceptual framework for understanding chronic pain in patients with HIV. *Pain Pract* 2014;**14**:207–16. doi:10.1111/papr.12052

3 Bridges S. Chapter 2: Mental health problem. In: *Health Survey*. 2014.

4 Abplanalp SJ, Mueser KT, Fulford D. The role of physical pain in global functioning of people with serious mental illness. *Schizophr Res* 2020;**222**:423–8. doi:10.1016/j.schres.2020.03.062

5 Public Health England. Severe mental illness (SMI) and physical health inequalities: briefing. Research and analysis. 2018.https://www.gov.uk/government/publications/severe-mental-illness-smi-physical-health-inequalities/severe-mental-illness-and-physical-health-inequalities-briefing#:~:text=The%20phrase%20severe%20mental%20illness,an%20SMI%20%5Bfootnote%201%5D. (accessed 23 Jun2023).

6 Stubbs B, Mitchell AJ, De Hert M, *et al.* The prevalence and moderators of clinical pain in people with schizophrenia: a systematic review and large scale meta-analysis. *Schizophr Res* 2014;**160**:1–8. doi:10.1016/j.schres.2014.10.017

7 Bonnot O, Anderson GM, Cohen D, *et al.* Are patients with schizophrenia insensitive to pain? A reconsideration of the question. *Clin J Pain* 2009;**25**:244–52. doi:10.1097/AJP.0b013e318192be97

8 Birgenheir DG, Ilgen MA, Bohnert ASB, *et al.* Pain conditions among veterans with schizophrenia or bipolar disorder. *Gen Hosp Psychiatry* 2013;**35**:480–4. doi:10.1016/j.genhosppsych.2013.03.019

9 Potvin S, Marchand S. Hypoalgesia in schizophrenia is independent of antipsychotic drugs: a systematic quantitative review of experimental studies. *Pain* 2008;**138**:70–8. doi:10.1016/j.pain.2007.11.007

10 IsHak WW, Wen RY, Naghdechi L, *et al.* Pain and depression: A systematic review. *Harv Rev Psychiatry* 2018;**26**:352–63. doi:10.1097/HRP.0000000000000198

11 Bair MJ, Robinson RL, Katon W, *et al.* Depression and pain comorbidity: a literature review. *Arch Intern Med* 2003;**163**:2433–45. doi:10.1001/archinte.163.20.2433

12 Blumer D, Heilbronn M. Chronic pain as a variant of depressive disease: the pain-prone disorder. *J Nerv Ment Dis* 1982;**170**:381–406. doi:10.1097/00005053-198207000-00001

13 Thompson T, Correll CU, Gallop K, *et al.* Is Pain Perception Altered in People With Depression? A Systematic Review and Meta-Analysis of Experimental Pain Research. *J Pain* 2016;**17**:1257–72. doi:10.1016/j.jpain.2016.08.007

14 Noroozian M, Raeesi S, Hashemi R, *et al.* Pain: the neglect issue in old people's life. *Open Access Maced J Med Sci* 2018;**6**:1773–8. doi:10.3889/oamjms.2018.335

15 Fillingim RB. Sex, gender, and pain. In: *Principles of Gender-Specific Medicine*. Elsevier 2017. 481–96. doi:10.1016/B978-0-12-803506-1.00038-3

16 Vallerand AH, Polomano RC. The relationship of gender to pain. *Pain Manag Nurs* 2000;**1**:8–15. doi:10.1053/jpmn.2000.9759

17 Campbell CM, Edwards RR. Ethnic differences in pain and pain management. *Pain Manag* 2012;**2**:219–30. doi:10.2217/pmt.12.7

18 Martin JL, McLean G, Park J, *et al.* Impact of socioeconomic deprivation on rate and cause of death in severe mental illness. *BMC Psychiatry* 2014;**14**:261. doi:10.1186/s12888-014-0261-4

19 Lambert TJR, Newcomer JW. Are the cardiometabolic complications of schizophrenia still neglected? Barriers to care. *Med J Aust* 2009;**190**:S39-42. doi:10.5694/j.1326-5377.2009.tb02374.x

20 DE Hert M, Correll CU, Bobes J, *et al.* Physical illness in patients with severe mental disorders. I. Prevalence, impact of medications and disparities in health care. *World Psychiatry* 2011;**10**:52–77. doi:10.1002/j.2051-5545.2011.tb00014.x

21 Frayne SM, Halanych JH, Miller DR, *et al.* Disparities in diabetes care: impact of mental illness. *Arch Intern Med* 2005;**165**:2631–8. doi:10.1001/archinte.165.22.2631

22 Laursen TM, Munk-Olsen T, Agerbo E, *et al.* Somatic hospital contacts, invasive cardiac procedures, and mortality from heart disease in patients with severe mental disorder. *Arch Gen Psychiatry* 2009;**66**:713–20. doi:10.1001/archgenpsychiatry.2009.61

23 Aro S, Aro H, Keskimäki I. Socio-economic mobility among patients with schizophrenia or major affective disorder. A 17-year retrospective follow-up. *Br J Psychiatry* 1995;**166**:759–67. doi:10.1192/bjp.166.6.759

24 Velupillai S, Suominen H, Liakata M, *et al.* Using clinical Natural Language Processing for health outcomes research: Overview and actionable suggestions for future advances. *J Biomed Inform* 2018;**88**:11–9. doi:10.1016/j.jbi.2018.10.005

25 Kadra G, Stewart R, Shetty H, *et al.* Long-term antipsychotic polypharmacy prescribing in secondary mental health care and the risk of mortality. *Acta Psychiatr Scand* 2018;**138**:123–32. doi:10.1111/acps.12906

26 Bendayan R, Kraljevic Z, Shaari S, *et al.* Mapping multimorbidity in individuals with schizophrenia and bipolar disorders: evidence from the South London and Maudsley NHS Foundation Trust Biomedical Research Centre (SLAM BRC) case register. *BMJ Open* 2022;**12**:e054414. doi:10.1136/bmjopen-2021-054414

27 Jackson RG, Patel R, Jayatilleke N, *et al.* Natural language processing to extract symptoms of severe mental illness from clinical text: the Clinical Record Interactive Search Comprehensive Data Extraction (CRIS-CODE) project. *BMJ Open* 2017;**7**:e012012. doi:10.1136/bmjopen-2016-012012

28 Sampson R, Cooper J, Barbour R, *et al.* Patients' perspectives on the medical primary-secondary care interface: systematic review and synthesis of qualitative research. *BMJ Open* 2015;**5**:e008708. doi:10.1136/bmjopen-2015-008708

29 Benchimol EI, Smeeth L, Guttmann A, *et al.* [The REporting of studies Conducted using Observational Routinely-collected health Data (RECORD) statement]. *Z Evid Fortbild Qual Gesundhwes* 2016;**115–116**:33–48. doi:10.1016/j.zefq.2016.07.010

30 von Elm E, Altman DG, Egger M, *et al.* Strengthening the Reporting of Observational Studies in Epidemiology (STROBE) statement: guidelines for reporting observational studies. *BMJ* 2007;**335**:806–8. doi:10.1136/bmj.39335.541782.AD

31 Stewart R, Soremekun M, Perera G, *et al.* The South London and Maudsley NHS Foundation Trust Biomedical Research Centre (SLAM BRC) case register: development and descriptive data. *BMC Psychiatry* 2009;**9**:51. doi:10.1186/1471-244X-9-51

32 N H S. Lambeth DataNet. Lambeth DataNet. 2021.https://selondonccg.nhs.uk/wp-content/uploads/2021/04/Lambeth-DataNet.pdf (accessed 26 May2023).

For peer review only

33 NIHR Maudsley Biomedical Research Center. CRIS Data Linkages. CRIS Data Linkages. https://www.maudsleybrc.nihr.ac.uk/facilities/clinical-record-interactive-search-cris/cris-data-linkages/ (accessed 7 Aug2023).

34 Fernandes AC, Cloete D, Broadbent MTM, *et al.* Development and evaluation of a de-identification procedure for a case register sourced from mental health electronic records. *BMC Med Inform Decis Mak* 2013;**13**:71. doi:10.1186/1472-6947-13-71

35 Research Design Service South Central. Patient and Public Involvement (PPI). What is Patient and Public Involvement in health and social care research? 2023.https://www.rds-sc.nihr.ac.uk/ppi-information-resources/

36 World Health Organization. International Classification of Diseases (ICD). 2008.https://www.who.int/standards/classifications/classification-of-diseases (accessed 7 Aug2023).

37 GOV.UK. English indices of deprivation 2019. 2019.https://www.gov.uk/government/statistics/english-indices-of-deprivation-2019 (accessed 7 Aug2023).

38 Chaturvedi J, Mascio A, Velupillai SU, *et al.* Development of a lexicon for pain. *Front Digit Health* 2021;**3**:778305. doi:10.3389/fdgth.2021.778305

39 Chaturvedi J, Velupillai S, Stewart R, *et al.* Identifying Mentions of Pain in Mental Health Records Text: A Natural Language Processing Approach. *arXiv* Published Online First: 2023. doi:10.48550/arxiv.2304.01240

40 NHS Digital. Read Codes. 2022.https://digital.nhs.uk/services/terminology-and-classifications/read-codes (accessed 7 Aug2023).

41 Ma R, Romano E, Ashworth M, *et al.* Multimorbidity clusters among people with serious mental illness: a representative primary and secondary data linkage cohort study. *Psychol Med* 2022;:1–12. doi:10.1017/S003329172200109X

42 NHSBSA. Dictionary of medicines and devices (dm+d) . 2023.https://www.nhsbsa.nhs.uk/pharmacies-gp-practices-and-appliance-contractors/dictionary-medicines-and-devices-dmd (accessed 2 Aug2023).

43 Barnett K, Mercer SW, Norbury M, *et al.* Epidemiology of multimorbidity and implications for health care, research, and medical education: a cross-sectional study. *Lancet* 2012;**380**:37–43. doi:10.1016/S0140-6736(12)60240-2

44 Outcomes Based Healthcare. Outcomes Platform. 2018.https://outcomesbasedhealthcare.com/outcomes-platform/ (accessed 3 Aug2023).

45 Hafezparast N, Bragan Turner E, Dunbar-Rees R, *et al.* Identifying populations with chronic pain in primary care: developing an algorithm and logic rules applied to coded primary care diagnostic and medication data. *BMC Primary Care* 2023.

46 Searle T, Kraljevic Z, Bendayan R, *et al.* MedCATTrainer: A Biomedical Free Text Annotation Interface with Active Learning and Research Use Case Specific Customisation. In: *Proceedings of the 2019 Conference on Empirical Methods in Natural Language Processing and the 9th International Joint Conference on Natural Language Processing (EMNLP-IJCNLP): System Demonstrations*. Stroudsburg, PA, USA: : Association for Computational Linguistics 2019. 139–44. doi:10.18653/v1/D19-3024

For peer review only

47 Perera G, Broadbent M, Callard F, *et al.* Cohort profile of the South London and Maudsley NHS Foundation Trust Biomedical Research Centre (SLaM BRC) Case Register: current status and recent enhancement of an Electronic Mental Health Record-derived data resource. *BMJ Open* 2016;**6**:e008721. doi:10.1136/bmjopen-2015-008721

48 Wandner LD, Scipio CD, Hirsh AT, *et al.* The perception of pain in others: how gender, race, and age influence pain expectations. *J Pain* 2012;**13**:220–7. doi:10.1016/j.jpain.2011.10.014

49 Fillingim RB. Sex, gender, and pain: women and men really are different. *Curr Rev Pain* 2000;**4**:24–30. doi:10.1007/s11916-000-0006-6

50 Green CR, Anderson KO, Baker TA, *et al.* The unequal burden of pain: confronting racial and ethnic disparities in pain. *Pain Med* 2003;**4**:277–94. doi:10.1046/j.1526-4637.2003.03034.x

51 Stubbs B, Thompson T, Acaster S, *et al.* Decreased pain sensitivity among people with schizophrenia: a meta-analysis of experimental pain induction studies. *Pain* 2015;**156**:2121–31. doi:10.1097/j.pain.0000000000000304

52 Samulowitz A, Gremyr I, Eriksson E, *et al.* “Brave Men” and “Emotional Women”: A Theory-Guided Literature Review on Gender Bias in Health Care and Gendered Norms towards Patients with Chronic Pain. *Pain Res Manag* 2018;**2018**:6358624. doi:10.1155/2018/6358624

53 Hoffmann DE, Tarzian AJ. The girl who cried pain: a bias against women in the treatment of pain. *J Law Med Ethics* 2001;**29**:13–27. doi:10.1111/j.1748-720X.2001.tb00037.x

54 American Psychiatric Association. Chronic Pain and Mental Health Often Interconnected. 2020.https://www.psychiatry.org/news-room/apa-blogs/chronic-pain-and-mental-health-interconnected (accessed 3 Aug2023).

55 Bennett C. What you need to know about pain and mental health in Australia. Pain Australia. 2020.https://www.painaustralia.org.au/media-document/blog-1/blog-2020/blog-2019/what-you-need-to-know-about-pain-and-mental-health-in-australia (accessed 3 Aug2023).

For peer review only

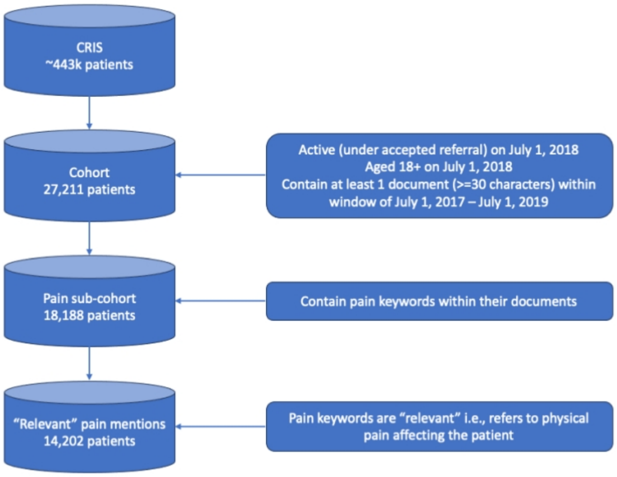

Figure 1. Data Extraction

339x191mm (102 x 102 DPI)

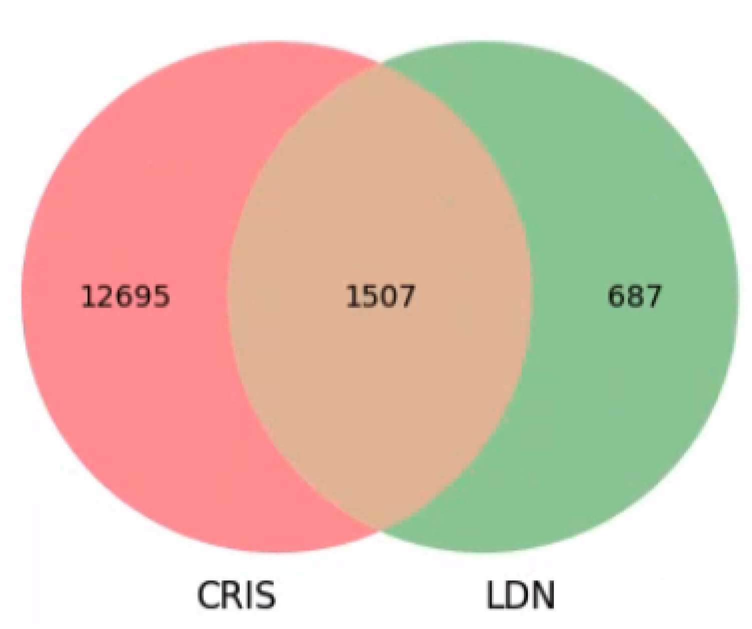

Figure 2. Overlap of recorded pain in CRIS and LDN

132x116mm (72 x 72 DPI)

The RECORD statement – checklist of items, extended from the STROBE statement, that should be reported in observational studies using routinely collected health data.

	Item No.	STROBE items	Location in manuscript where items are reported	RECORD items	Location in manuscript where items are reported
**Title and abstract**					
	1	(a) Indicate the study's design with a commonly used term in the title or the abstract (b) Provide in the abstract an informative and balanced summary of what was done and what was found	(a) Title<br>(b) Abstract	RECORD 1.1: The type of data used should be specified in the title or abstract. When possible, the name of the databases used should be included.<br><br>RECORD 1.2: If applicable, the geographic region and timeframe within which the study took place should be reported in the title or abstract.<br><br>RECORD 1.3: If linkage between databases was conducted for the study, this should be clearly stated in the title or abstract.	Abstract
**Introduction**					
Background rationale	2	Explain the scientific background and rationale for the investigation being reported	Introduction - Background Rationale		
Objectives	3	State specific objectives, including any prespecified hypotheses	Introduction - Objectives		
**Methods**					
Study Design	4	Present key elements of study design early in the paper	Methods		
Setting	5	Describe the setting, locations, and relevant dates, including periods of recruitment, exposure, follow-up, and data collection	Methods - Setting and Variables		

For peer review only

Participants	6	*(a) Cohort study* - Give the eligibility criteria, and the sources and methods of selection of participants. Describe methods of follow-up<br>*Case-control study* - Give the eligibility criteria, and the sources and methods of case ascertainment and control selection. Give the rationale for the choice of cases and controls<br>*Cross-sectional study* - Give the eligibility criteria, and the sources and methods of selection of participants<br><br>*(b) Cohort study* - For matched studies, give matching criteria and number of exposed and unexposed<br>*Case-control study* - For matched studies, give matching criteria and the number of controls per case	Methods - Participants	RECORD 6.1: The methods of study population selection (such as codes or algorithms used to identify subjects) should be listed in detail. If this is not possible, an explanation should be provided.<br><br>RECORD 6.2: Any validation studies of the codes or algorithms used to select the population should be referenced. If validation was conducted for this study and not published elsewhere, detailed methods and results should be provided.<br><br>RECORD 6.3: If the study involved linkage of databases, consider use of a flow diagram or other graphical display to demonstrate the data linkage process, including the number of individuals with linked data at each stage.	
Variables	7	Clearly define all outcomes, exposures, predictors, potential confounders, and effect modifiers. Give diagnostic criteria, if applicable.	Methods - Variables	RECORD 7.1: A complete list of codes and algorithms used to classify exposures, outcomes, confounders, and effect modifiers should be provided. If these cannot be reported, an explanation should be provided.	
Data sources/ measurement	8	For each variable of interest, give sources of data and details of methods of assessment (measurement).<br>Describe comparability of assessment methods if there is more than one group	Methods - Variables		

For peer review only

Bias	9	Describe any efforts to address potential sources of bias	Methods - Variables		
Study size	10	Explain how the study size was arrived at	Methods - Participants		
Quantitative variables	11	Explain how quantitative variables were handled in the analyses. If applicable, describe which groupings were chosen, and why	Methods - Variables		
Statistical methods	12	(a) Describe all statistical methods, including those used to control for confounding<br>(b) Describe any methods used to examine subgroups and interactions<br>(c) Explain how missing data were addressed<br>(d) *Cohort study* - If applicable, explain how loss to follow-up was addressed<br>*Case-control study* - If applicable, explain how matching of cases and controls was addressed<br>*Cross-sectional study* - If applicable, describe analytical methods taking account of sampling strategy<br>(e) Describe any sensitivity analyses	Methods - Setting and Descriptive Statistics		
Data access and cleaning methods		..		RECORD 12.1: Authors should describe the extent to which the investigators had access to the database population used to create the study population.	Methods - Setting - Ethical Approval

For peer review only

				RECORD 12.2: Authors should provide information on the data cleaning methods used in the study.	Methods - Variables
Linkage		..		RECORD 12.3: State whether the study included person-level, institutional-level, or other data linkage across two or more databases. The methods of linkage and methods of linkage quality evaluation should be provided.	Methods - Setting
**Results**					
Participants	13	(a) Report the numbers of individuals at each stage of the study (*e.g.*, numbers potentially eligible, examined for eligibility, confirmed eligible, included in the study, completing follow-up, and analysed)<br>(b) Give reasons for non-participation at each stage.<br>(c) Consider use of a flow diagram	Results	RECORD 13.1: Describe in detail the selection of the persons included in the study (*i.e.*, study population selection) including filtering based on data quality, data availability and linkage. The selection of included persons can be described in the text and/or by means of the study flow diagram.	Methods
Descriptive data	14	(a) Give characteristics of study participants (*e.g.*, demographic, clinical, social) and information on exposures and potential confounders<br>(b) Indicate the number of participants with missing data for each variable of interest<br>(c) *Cohort study* - summarise follow-up time (*e.g.*, average and total amount)	Results		
Outcome data	15	*Cohort study* - Report numbers of outcome events or summary measures over time<br>*Case-control study* - Report numbers in each exposure	Results		

For peer review only

		category, or summary measures of exposure<br>*Cross-sectional study* - Report numbers of outcome events or summary measures			
Main results	16	(a) Give unadjusted estimates and, if applicable, confounder-adjusted estimates and their precision (e.g., 95% confidence interval). Make clear which confounders were adjusted for and why they were included<br>(b) Report category boundaries when continuous variables were categorized<br>(c) If relevant, consider translating estimates of relative risk into absolute risk for a meaningful time period	Results		
Other analyses	17	Report other analyses done—e.g., analyses of subgroups and interactions, and sensitivity analyses	Results		
**Discussion**					
Key results	18	Summarise key results with reference to study objectives	Discussion		
Limitations	19	Discuss limitations of the study, taking into account sources of potential bias or imprecision. Discuss both direction and magnitude of any potential bias	Discussion	RECORD 19.1: Discuss the implications of using data that were not created or collected to answer the specific research question(s). Include discussion of misclassification bias, unmeasured confounding, missing data, and changing eligibility over time, as they pertain to the study being reported.	Discussion and Conclusion
Interpretation	20	Give a cautious overall interpretation of results considering objectives,			

For peer review only

		limitations, multiplicity of analyses, results from similar studies, and other relevant evidence	Discussion		
Generalisability	21	Discuss the generalisability (external validity) of the study results	Discussion		
**Other Information**					
Funding	22	Give the source of funding and the role of the funders for the present study and, if applicable, for the original study on which the present article is based	Acknolwledgements and disclosure of interests		
Accessibility of protocol, raw data, and programming code		..	Data Availability Statement	RECORD 22.1: Authors should provide information on how to access any supplemental information such as the study protocol, raw data, or programming code.	Data Availability Statement

*Reference: Benchimol EI, Smeeth L, Guttmann A, Harron K, Moher D, Petersen I, Sørensen HT, von Elm E, Langan SM, the RECORD Working Committee. The REporting of studies Conducted using Observational Routinely-collected health Data (RECORD) Statement. *PLoS Medicine* 2015; in press.

*Checklist is protected under Creative Commons Attribution (CC BY) license.

For peer review only

BMJ Open

Distributions of Recorded Pain in Mental Health Records: A Natural Language Processing Based Study

Journal:	*BMJ Open*
Manuscript ID	bmjopen-2023-079923.R1
Article Type:	Original research
Date Submitted by the Author:	01-Feb-2024
Complete List of Authors:	Chaturvedi, Jaya; King's College London, Department of Biostatistics and Health Informatics<br>Stewart, Robert; King's College London, Institute of Psychiatry<br>Ashworth, Mark; King's College London, School of Population Health & Environmental Sciences<br>Roberts, Angus; King's College London, Institute of Psychiatry, Psychology and Neuroscience
**Primary Subject Heading**:	Health informatics
Secondary Subject Heading:	Epidemiology, Mental health
Keywords:	Natural Language Processing, Electronic Health Records, EPIDEMIOLOGY, MENTAL HEALTH, Chronic Pain

SCHOLARONE™
Manuscripts

I, the Submitting Author has the right to grant and does grant on behalf of all authors of the Work (as defined in the below author licence), an exclusive licence and/or a non-exclusive licence for contributions from authors who are: i) UK Crown employees; ii) where BMJ has agreed a CC-BY licence shall apply, and/or iii) in accordance with the terms applicable for US Federal Government officers or employees acting as part of their official duties; on a worldwide, perpetual, irrevocable, royalty-free basis to BMJ Publishing Group Ltd ("BMJ") its licensees and where the relevant Journal is co-owned by BMJ to the co-owners of the Journal, to publish the Work in this journal and any other BMJ products and to exploit all rights, as set out in our licence.

The Submitting Author accepts and understands that any supply made under these terms is made by BMJ to the Submitting Author unless you are acting as an employee on behalf of your employer or a postgraduate student of an affiliated institution which is paying any applicable article publishing charge ("APC") for Open Access articles. Where the Submitting Author wishes to make the Work available on an Open Access basis (and intends to pay the relevant APC), the terms of reuse of such Open Access shall be governed by a Creative Commons licence – details of these licences and which Creative Commons licence will apply to this Work are set out in our licence referred to above.

Other than as permitted in any relevant BMJ Author's Self Archiving Policies, I confirm this Work has not been accepted for publication elsewhere, is not being considered for publication elsewhere and does not duplicate material already published. I confirm all authors consent to publication of this Work and authorise the granting of this licence.

For peer review only

Distributions of Recorded Pain in Mental Health Records: A Natural Language Processing Based Study

Jaya Chaturvedi[1*], Robert Stewart[1,2], Mark Ashworth[1], Angus Roberts[1]
[1]Institute of Psychiatry, Psychology and Neurosciences, King's College London
[2]South London and Maudsley NHS Foundation Trust
*Corresponding author: jaya.1.chaturvedi@kcl.ac.uk

For peer review only

Abstract

Objective

The objective of this study is to determine demographic and diagnostic distributions of physical pain recorded in clinical notes of a mental health electronic health records database by utilising natural language processing and to examine the overlap in recorded physical pain between primary and secondary care.

Design, Setting and Participants

The data were extracted from an anonymised version of the electronic health records from a large secondary mental healthcare provider serving a catchment of 1.3M residents in south London. These included patients under active referral, aged 18+ at index date of July 1, 2018, and had at least one clinical document (>=30 characters) between July 1, 2017 and July 1, 2019. This cohort was compared to linked primary care records from one of the four local government areas.

Outcome

The primary outcome of interest was the presence of recorded physical pain within the clinical notes of the patients, not including psychological or metaphorical pain.

Results

A total of 27,211 patients were retrieved. Of these, 52% (14,202) had narrative text containing relevant mentions of physical pain. Patients who were older (OR 1.17, 95% CI 1.15-1.19), female (OR 1.42, 95% CI 1.35-1.49), Asian (OR 1.30, 95% CI 1.16-1.45) or Black (OR 1.49, 95% CI 1.40-1.59) ethnicities, living in deprived neighbourhoods (OR 1.64, 95% CI 1.55-1.73) showed higher odds of recorded pain. Patients with severe mental illnesses were found to be less likely to report pain (OR 0.43, 95% CI 0.41-0.46, p<0.001). 17% of the cohort from secondary care also had records within primary care.

Conclusion

The findings of this study show the sociodemographic and diagnostic differences in recorded pain. Specifically, lower documentation across certain groups indicates needs for better screening protocols and training on recognising varied pain presentations. Additionally, targeting improved detection for minority and disadvantaged groups can promote health equity.

Keywords: Natural Language Processing, Pain, Mental Health, Electronic Health Records

Word count: 4,880

Strengths and Limitations of this study

- This study utilises natural language processing on clinical notes to access a large sample with information about pain. The identification of such information would not be feasible manually
- This is the first cross-sectional study to summarise and describe the distribution of recorded pain within the clinical notes of mental health records.
- Inclusion of both secondary mental health and primary care records for the same patients allows comparison of pain documentation across different health services.
- When patients show no recorded pain, the study does not differentiate between pain that was discussed but not recorded, or pain that was not discussed.
- The findings are not generalisable to the general population since this study only looks at patients receiving mental healthcare within a specific geographic catchment.

Introduction

Background Rationale

Pain and its relationship with mental health are important research topics. Pain has imposed a significant burden on society in terms of medical care costs as well as lost productivity [1,2]. Pain is multifaceted, with physical, psychological, social, and biological causes and consequences [3,4]. Mental health disorders also present a considerable and complex public health problem, being a leading cause of disability and accounting for 28% of the national disease burden in the UK [5]. Electronic health records (EHRs) for mental health are a significant source of information for studying the intersection between pain and mental health within those who receive specialist service input. EHRs open the possibility of investigating how pain is recorded and its impact on clinical outcomes.

Severe mental illnesses (SMIs) include diagnoses of schizophrenia spectrum disorder, bipolar disorder, or severe major depressive disorder [6], where functional and occupational activities are severely impaired due to associated debilitating psychological problems [7]. While several studies have looked at the relationship between pain and schizophrenia and bipolar disorders [8–11] and at other mental illnesses such as depression [12–16], the complex and potentially bidirectional nature of this relationship requires further understanding. Analysis of secondary

For peer review only

data sources, such as EHR databases, might help by providing a fuller picture of the recorded clinical presentation of this group of patients; however, a prerequisite is that pain is adequately represented in derived data.

Demographic features such as age, gender and ethnicity may affect how pain is perceived and experienced. Pain affects twice as many persons over the age of 60 as it does younger individuals [17]. While pain is not a natural feature of the ageing process, many health conditions causing pain become more common with increasing age. Nonetheless, older patients often believe pain to be a normal aspect of ageing and might be hesitant when reporting it [17]. There have also been variations found in the reported perception of pain by female and male patients, with female patients reporting experiencing more pain than males [18,19]. Research has also shown disparities in pain perception across different ethnicities, with individuals of Black African ethnicity reporting greater pain than White counterparts [20].

Socioeconomic status (SES) plays a role in health and overall well-being, with deprivation associated with unfavourable health outcomes and increased mortality rates [21]. Patients with SMI already experience higher mortality rates than the general population, and this discrepancy is exacerbated by socioeconomic deprivation, primarily due to unequal access to good quality physical healthcare services [22–25]. Furthermore, patients with SMI continue to experience a decline in their SES over time, compounding its impact [26]. Given these well-established connections between lower SES and reduced access to care [27,28], examination of potential SES-based differences in documentation of physical symptoms such as pain is particularly relevant. As disadvantaged patients face barriers in the screening for comorbid conditions, this may manifest in lower rates of discussions and recording of pain symptoms.

Most patient information is recorded in unstructured clinical narratives within EHR databases [29], and pain is likely to be no different, with few, if any, structured checklists ascertaining its presence in routine clinical care. Natural language processing (NLP), a computational approach to understanding and analysing human language, is therefore potentially useful for extracting such pain information. NLP has been applied extensively to EHR data, including studies of SMI, such as antipsychotic polypharmacy in mental health care [30], multimorbidity in individuals with schizophrenia and bipolar disorders [31], and extracting symptoms of SMI [32].

In addition to secondary care data, it is also useful to consider the recording of pain in primary care data. Within the UK, primary care (GP - general practice) is generally the first point of contact for patients [33]. Exploring the overlap of recorded pain between primary care GP services and secondary care mental health services could, therefore, provide a more comprehensive view of the patient's pain experiences, and any discrepancies could highlight gaps in care and communication across different parts of the healthcare system. As primary care physicians are often responsible for the initial pain assessment and referral to specialists if needed, documentation patterns in GP records versus mental health provider records may differ for those with psychiatric disorders. Comparing recorded pain rates across these care settings can reveal insights into the consistency of pain screening among this vulnerable population across the healthcare landscape. This study utilised GP records specifically for patients contained within a mental health secondary care database in order to explore documentation patterns in primary care for patients who were recorded in secondary care with a mental illness. Analysis of these GP records takes the documentation beyond specialist

For peer review only

mental health settings, and provides valuable insights into the larger healthcare experiences of those with mental health disorders. Additionally, examination of potential differences or overlap between the primary and secondary care for the same patient cohort enables important observations about consistency of pain assessment across providers.

Objectives

The objective of this study is to describe the distributions of recorded pain amongst mental health service users according to demographic factors such as age, gender and ethnicity, as well as neighbourhood deprivation levels and mental health diagnoses. This was achieved by examining recorded pain through the means of an NLP application within the clinical text of a mental health EHR database, and further evaluating this by measuring the overlap between pain recorded in secondary and primary health care, enabled through data linkage between the two.

This research aims to address knowledge gaps regarding the documentation of pain among mental health populations. In particular, a clearer understanding of these patterns is essential given the exceptionally high rates of pain conditions comorbid with mental health disorders. This study will answer fundamental questions around the frequencies of documented pain discussions during mental health encounters, consistency in pain detection across primary vs secondary care settings, and whether certain groups defined by gender, ethnicity or socioeconomic status face greater gaps in pain inquiry documentation. By analysing rates and differences of recorded pain within mental health records using a population-based cohort, this study works towards addressing needs around appropriate pain identification as a routine component of comprehensive mental health treatment.

Methods

Reporting

We use the RECORD [34] guidelines and checklist, an extension of the STROBE [35] guidelines, for reporting the results of this study.

Setting

Data on recorded pain, which in this context refers to any mentions of physical pain within the clinical notes, such as “complains of pain” and “experiencing headaches”, were obtained from the clinical text of a mental health EHR database, the Clinical Record Interactive Search (CRIS) resource. This contains a de-identified version of EHR data from The South London and Maudsley NHS Foundation Trust (SLaM), one of Europe's largest mental healthcare organisations [36], which serves a geographic catchment of around 1.3 million residents in four south London boroughs (Croydon, Lambeth, Lewisham, Southwark). CRIS contains about 30 million free text documents, averaging 90 documents per patient [29].

For peer review only

Data were also obtained from a primary care database called Lambeth DataNet (LDN) [37], which accesses all GP records from general practices based in the London borough of Lambeth. Data linkages (at the patient level) are already in place between CRIS and LDN [38].

Ethical Approval

CRIS (as well as its associated linkages) has received ethical approval as a data resource for secondary analysis from the Oxford C Research Ethics Committee (reference 23/SC/0257). A patient-led oversight committee (detailed in [39]) reviews and approves research projects that use the CRIS database. For service users, an opt-out system is in place and is advertised in all promotional materials and campaigns. Only authorised individuals can access this data from within a secure firewall. The CRIS project approval references for this work are 21-021 and 23-003.

LDN approval was obtained as part of an existing CRIS project (project number 23-124) which included access to linked data from LDN (Caldicott Guardian approval, 15/9/21). This CRIS-LDN project aimed to examine the profile of patients with mental illnesses and chronic/persistent pain and compare them to controls from LDN who had chronic/persistent pain only.

Patient and Public Involvement

Patient and public involvement (PPI) in research is an active collaboration between researchers and members of the public, where the latter actively participate in contributing to the research [40]. A PPI group with lived experiences of SMI and chronic pain were consulted as part of this research. The nature of the data available was described to the group, and they were asked about their priorities regarding what research questions they would like answered. In response to this, the group was unanimously interested in further study of the differences in pain experiences based on demographics and diagnoses, and this was the main motivation for the objective of the study described here.

Participants

A cohort of patients was extracted from the CRIS database comprising those who were active (i.e., the secondary care hospital trust (SLaM) has accepted them as a referral) and aged 18+ on the index date of July 1, 2018, and whose record contained at least one document (>= 30 characters) within a window of July 1, 2017 to July 1, 2019.

LDN extraction followed similar criteria for patients who were active on the index date, aged 18+, and contained pain diagnoses or medications from July 1, 2017 to July 1, 2019. Free-text information is unavailable within LDN, so no document criteria were required.

For peer review only

Variables

Demographics

Age, gender, and ethnicity variables were extracted from structured tables within the CRIS database. Individuals with missing ethnicity values were retained as a separate category (Not stated/known).

Diagnosis

The primary diagnosis recorded closest to the index date of July 1, 2018, was extracted from the structured tables within the CRIS database. These are coded using ICD-10 [41]. The diagnosis codes were categorised as SMI and non-SMI, where SMI includes ICD-10 codes of F20-29 and F30-33.

Deprivation

The Index of Multiple Deprivation (IMD) decile measures from 2019 [42] were extracted for information on neighbourhood deprivation for each patient, based on their address at the time of the index date aggregated by Lower Super Output Area (LSOA) - a standard national administrative unit containing an average 1500 residents. National Census data are used to calculate IMD scores for each LSOA. A lower IMD decile indicates higher deprivation levels. Individuals with missing IMD scores were retained in a separate category.

Recorded Pain

Pain-related keywords generated from a lexicon of pain terms [43] were used to identify patients in the cohort who had mentions of physical pain (such as “worsening back pain”, “suffers from headaches”, “complains of pain”) recorded in their clinical notes within the predetermined window. The lexicon contains terms such as aching muscles, backpain, headache, myalgia, etc and can be accessed online[1]. An NLP application (F1-score, i.e., harmonic mean of precision and recall: 0.98) was used on the documents of these patients. The application was developed by fine-tuning an existing model (SapBERT [44]) with 5,644 gold standard annotations (triple annotated by medical student annotators) from CRIS, with the intention of classifying sentences within documents as relevant or not, where relevant refers to a mention of physical pain affecting the patient, such as “complained of pain”, “has muscle aches..”, and not relevant refers to no or negated mentions, hypothetical mentions, and metaphorical mentions of pain, such as “..defensive of painful feelings”, and “..painful consequences of using alcohol”. Only relevant mentions were used in the results reported here. The application has been described in detail in [45].

As with all other UK research based on access to anonymised primary care records, LDN does not allow access to any free text clinical notes. For this reason, pain information can only be extracted from the structured fields of the records. Read codes [46] were used to identify patients who had a pain diagnosis or were on any pain medications and treatments:

[1]https://docs.google.com/spreadsheets/d/1z-6619UBdvwWrB9Sz4b1rbjDzuslOGCpts2DNc0naCc/edit?usp=sharing

For peer review only

1. Pain medications code list - developed as part of a project described in [47], which focused on analgesics (obtained from dm+d (a dictionary of medicines and devices [48]) used in the treatment of 35 long-term conditions. These 35 conditions were obtained from [49], a cross-sectional study on multimorbidities in patients registered with 314 medical practices in Scotland as of March 2007.
2. Pain diagnosis and treatments code list - developed as part of a collaboration project with Outcomes Based Healthcare (OBH), an organisation that provides a platform for the study of population health outcomes [50], with the research described in [51]. Pain diagnosis codes included instances such as back pain, referred ear pain, arthritis, and trigeminal neuralgia. Pain treatment codes included codes for referral to pain clinic, seen in pain clinic, and under the care of pain management specialist.

While these codes were developed for chronic pain, they are generic enough to be used for this research, as highlighted in the examples mentioned. These code lists are available on GitHub[2].

Anatomy Related to Recorded Pain

Another NLP application was developed as part of this research for identifying anatomy mentioned in relation to pain. This was a sentence classifier that generated a binary output - “mentioned” or “not mentioned”. The application was trained on 4,026 gold standard sentences about anatomy mentioned in relation to pain, and performed with an F1 score of 0.94. These gold standard sentences were a subset of the sentences used to train the pain NLP application. This application was run on sentences labelled as relevant by the pain application. Once the sentences that contained mentions of body parts were identified, they were run through MedCATTrainer [52], which used named entity recognition (NER), a type of NLP task to label entities within the text to identify the specific body parts mentioned within the text. The purpose of using MedCATTrainer was that it linked the identified body parts to unique identification numbers (SCTID) from SNOMED CT, a terminology of clinical terms. These SCTIDs were used to aggregate the mentioned body parts, for ease of analysis. For example, foot, calf, and knee mentions would be aggregated under “lower limb”.

Overlap between CRIS and LDN

To examine the overlap across primary (LDN) and secondary (CRIS) care, the patient IDs from the CRIS cohort (N=27,211) were searched for matching records within the LDN database over the same window of July 1, 2017 to July 1, 2019. Variables were generated indicating the presence of the patients within LDN, along with variables indicating the presence of any codes for pain medication, diagnosis or treatment based on the predefined lists described above. This allowed the identification of patients with documented pain experiences in both their mental health and primary care records for the aligned time period. The cross-referencing process enabled the comparison of recorded pain between the two systems at the patient level.

[2] https://github.com/jayachaturvedi/pain_in_mental_health

For peer review only

Descriptive Statistics

All analysis was conducted using STATA v15.1 and the Python programming language (version 3.10.0).

Descriptive statistics were obtained for demographic, deprivation and diagnosis features and compared between the two groups - patients who had recorded pain (class 1) and those who did not (class 0) - within their clinical notes. Chi-squared tests and logistic regression were conducted between the two classes to obtain adjusted odds ratios. Frequencies of body parts affected by pain and the overlap of recorded pain experiences between CRIS and LDN were also reported.

Results

Data Extraction

Based on the extraction criteria, 27,211 patients were represented. Amongst these patients, 18,188 had pain keywords mentioned within their documents. These documents were run through the NLP application to label them as relevant to pain (class 1) or not (class 0), resulting in 14,202 patients who had relevant mentions of pain within their clinical notes (Figure 1). Relevant mentions include instances such as "complains of pain", "experiencing headaches", "worsening back pain", and "has stomach cramps". Mentions that were not relevant were instances such as negations ("denied pain..", "no complaint of pain"), mentions within forms ("..experiencing other physical symptoms? E.g. chest pain", misspelt words ("..pained and decorated the walls"), hypothetical mentions (" reduce risk of pressure sores", "fear that eating will cause throat pain"), and metaphorical mentions ("life is too painful to carry on living", "pain will end when she repents").

<Figure 1>

Cohort Characteristics and Pain Mentions

Amongst the cohort of 27,211 patients, the mean age of the cohort was 44 (Inter-quartile range 29-55, SD 17.5), with 50.3% female and 48.2% of White ethnicity. The majority of the cohort (72.2%) lived in more deprived areas (IMD score <=5), and 67.0% received a non-SMI diagnosis. 66.8% of the patients (18,188 patients and 174,167 mentions within documents) contained pain keywords within their documents, and 52.1% of the cohort (14,202 patients) contained relevant mentions of pain in their documents.

Records of 52.1% of the patients within the cohort contained relevant mentions of pain. Differences between the patients who showed recorded pain (class 1) in their clinical notes and those who didn't (class 0) are shown in Table 1. Class 0 includes patients who did not have any pain mentions in their documents, as well as patients whose pain mentions were classified as not relevant. Patients within class 1 had a mean of10 pain mentions per document (median = 4).

For peer review only

Characteristic	n	Class 0 (no recorded pain)	Class 1 (recorded pain)
N (%)	27,211	13,009 (47.9)	14,202 (52.1)
Mean Age (IQR)	44 (29–55)	41 (27–52)	46 (32–56)
Gender (N, %)			
Male	13,471	7,037 (54.1)	6,434 (45.3)
Female	13,709	5,953 (45.7)	7,756 (54.6)
Not known	31	19 (0.2)	12 (0.1)
Ethnicity (N, %)			
White	13,139	6,014 (46.2)	7,125 (50.1)
Black	5,866	2,115 (16.2)	3,751 (26.4)
Not stated/known	4,708	3,418 (26.2)	1,290 (9.0)
Asian	1,506	592 (4.5)	914 (6.4)
Other	1,197	512 (3.9)	685 (4.8)

For peer review only

Mixed	795	358 (2.7)	437 (3.0)
Index of Multiple Deprivation (N, %) Decile 2019			
<= 5 (more deprived)	19,660	8,847 (68.0)	10,813 (76.1)
> 5 (less deprived)	6,686	3,836 (29.4)	2,850 (20.0)
Not known	865	326 (2.5)	539 (3.9)
Primary Diagnosis: SMI vs Non-SMI (ICD-9 code) (N, %)			
SMI	8,962	3,059 (23.5)	5,903 (41.5)
Non-SMI	18,249	9,950 (76.5)	8,299 (58.5)

For peer review only

Table 1. Distributions between the two classes - class 0 (no recorded pain) and class 1 (recorded pain)

Demographic variations emerged between those with/without recorded pain in the cohort, as shown in Table 1. The mean age was higher in patients with recorded pain at 46 (SD=17) compared to 41 (SD=17) for the remainder. Patients with recorded pain were more likely to be female and had a higher representation across all ethnic minorities. Additionally, patients with documented pain experiences were more likely to live in higher deprivation neighbourhoods.

Table 2 presents demographic, deprivation and diagnostic associations with recorded pain obtained through logistic regressions (unadjusted and adjusted for different factors as detailed below).

	Logistic Regression models				
	Unadjusted	Mutually adjusted			
		Model 1	Model 2	Model 3	Model 4
**Age** (per 10 years)	1.17 [1.15, 1.19] *	1.12 [1.11, 1.14] *	1.12 [1.11, 1.14] *	1.11 [1.10, 1.13] *	-

**Gender**					
Male	1 (reference)	1 (reference)	1 (reference)	1 (reference)	-
Female	1.42 [1.35, 1.49] *	1.42 [1.35, 1.49] *	1.43 [1.36, 1.50] *	1.47 [1.40, 1.55] *	-
Not known	0.69 [0.33, 1.42]	1.08 [0.50, 2.33]	1.06 [0.49, 2.30]	1.10 [0.51, 2.38]	-
**Ethnicity**					
White	1 (reference)	1 (reference)	1 (reference)	1 (reference)	1 (reference)
Asian	1.30 [1.16, 1.45] *	1.36 [1.22, 1.52] *	1.34 [1.19, 1.49] *	1.21 [1.08, 1.36] *	1.29 [1.15, 1.44] *
Black	1.49 [1.40, 1.59] *	1.58 [1.48, 1.69] *	1.50 [1.40, 1.60] *	1.25 [1.17, 1.34]	1.42 [1.33, 1.52] *
Other	1.12 [1.00, 1.27]	1.20 [1.06, 1.36]	1.17 [1.03, 1.32]	1.10 [0.97, 1.24]	1.08 [0.96, 1.33]
Mixed	1.03 [0.89, 1.18]	1.15 [0.99, 1.33]	1.12 [0.96, 1.30]	1.06 [0.91, 1.23]	1.01 [0.87, 1.17]
Not known	0.31 [0.29, 0.34] *	0.36 [0.34, 0.39] *	0.37 [0.34, 0.40] *	0.40 [0.37, 0.44] *	0.32 [0.30, 0.35] *
**Index of Multiple Deprivation**					
National Decile <=5	1.64 [1.55, 1.73] *	-	1.43 [1.35, 1.51] *	1.37 [1.29, 1.45] *	1.41 [1.33, 1.50] *
**Diagnosis**					
SMI	0.43 [0.41, 0.46] *	-	-	0.56 [0.53, 0.59] *	-

Table 2. Logistic Regression findings for variables reflecting differences in class 0 (no recorded pain) and class 1 (recorded pain) (N = 27,211). Outcome is recorded or no recorded pain.
Values are given as odds ratio (95% CI), and * indicates significance at $p < 0.001$
Model 1 contained the demographic variables only [age, gender and ethnicity].
Model 2 contained the variables from Model 1, plus the variable for deprivation (IMD Decile].
Model 3 contained the variables from Model 2 plus the diagnosis variable.
Model 4 contains the ethnicity and deprivation variables alone.

Unadjusted odds ratios revealed patients with documented pain experiences were more likely to be older (OR 1.17, 95% CI 1.15-1.19, p<0.001), female (OR 1.42, 95% CI 1.35-1.49, p<0.001), of Asian (OR 1.30 in relation to a White reference group, 95% CI 1.16-1.45, p<0.001) or Black (OR 1.49, 95% CI 1.40-1.59, p<0.001) ethnicities, and living in deprived neighbourhoods (OR 1.64, 95% CI 1.55-1.73, p<0.001) when compared to the respective reference groups. In a model containing all demographic variables (Model 1), the odds ratios for documented pain were higher for all ethnic minority groups compared to the White group. With additional adjustment for neighbourhood deprivation (Model 2), the odds ratios increased

For peer review only

further for females relative to males. In the model also adjusted for diagnoses (Model 3), the odds ratios were also higher for females versus males. Patients with SMI had lower odds of documented pain (OR 0.43, 95% CI 0.41-0.46, p<0.001) than non-SMI patients, which slightly reduced after accounting for demographics, deprivation and diagnosis (Model 3). A supplementary model (Model 4) including both ethnicity and deprivation as covariates showed increased odds for Asian and Black ethnicities when compared to White patients, and those in more deprived neighbourhoods. The motivation for this model was to disentangle the independent contributions of ethnicity and deprivation to the differences in pain documentation. By adjusting for deprivation while evaluating the association between ethnicity and recorded pain (and vice versa), we can derive better effect estimates for each factor. This approach helps us to understand whether ethnicity-related differences persist after accounting for socioeconomic factors. We present selected incremental models for transparency in how estimates shifted with inclusion of covariates, but focus our interpretation on the unadjusted and fully adjusted model 3, which highlight the patterns with most clarity.

Anatomy Distributions

Additional descriptive data were generated on the anatomical location of the pain reported. Amongst the 14,202 patients with any recorded pain, there were 174,167 mentions of pain within the documents. Of these, 7,555 (53%) patients included 40,418 mentions of the anatomy associated with the pain. Of these 53%, each patient had an average of 5 body parts mentioned in the context of pain. The most common body part affected by pain, as per the recorded mentions, was lower limbs, which accounted for 20% of all mentions where anatomy could be ascertained (Table 3).

**Body Part**	**Mentions**	**Frequency (mention-level)**
Lower limbs	Feet, ankle, leg, knee, calf, thigh, toes	20%
Upper body, excluding back	Chest, side of chest, upper body, torso	19%
Upper limbs	Hand, wrist, arm, elbow, thumb, shoulder	17%
Stomach/abdomen region	Stomach, abdomen, groin, bladder, prostate	16%
Head and neck	Head, tooth, face, mouth, tongue, eye, ear, neck	15%
Non-specific site	Entire body, skin, muscle, joint	8%

Back	Back, lower back	5%

Table 3. Body parts affected (at mention level)

Similar distributions were found within the SMI and non-SMI groups. Amongst patients with an SMI diagnosis, the most frequent body parts mentioned were lower limbs (23%), upper body, excluding back (17%) and stomach/abdomen region (15%). Patients with a non-SMI diagnosis most frequently reported lower limbs (19%), stomach/abdomen region (18%) and upper body, excluding back (17%), with minor variations in the frequencies.

Overlap with Primary Care

When comparing secondary care CRIS records with those of primary care from LDN, among the 27,211 patients of the CRIS cohort, 4,822 patients (17%) also had records in LDN. Amongst these patients who had records in both CRIS and LDN, 1,507 (31%) patients were identified as having some recorded instance of pain in both their records, while 687 (14%) patients showed recorded pain only in LDN (primary care). Among the 27,211 patients within CRIS, 12,695 (46%) had recorded pain only within CRIS (mental health care), as seen in Figure 2.

<Figure 2>

Discussion

This study investigated the differences observed in recorded pain mentions within the clinical notes of mental health records. The results reflect current literature findings that pain is a common issue among patients with mental health disorders. In a cohort of 27,211 patients, 18,188 (67%) patients contained pain-related keywords in their text, and 14,202 (52%) patients had relevant pain mentions, i.e., the mention indicated physical pain affecting the patient in question. We found differences in documented pain mentions across genders, with a greater proportion recorded among female patients. This aligns with previous literature demonstrating gender differences in pain reporting and experiences [16,48,49,53,54]. Furthermore, while patients with known ethnicities had higher frequencies of recorded pain in the cohort of relevant pain mentions (in relation to those with unknown ethnicity), most noticeable were the Black, Asian and other ethnic groups. This highlights the need for a comprehensive exploration of pain experiences across diverse populations [55]. Moreover, the study's findings are also consistent with studies that indicate the impact of deprivation on health outcomes [21], as people living in more deprived areas (IMD decile <= 5) were more frequently recorded with pain.

When comparing the overlap of patients between primary and secondary care, it was found that 17% of the patients within the CRIS cohort also had records within LDN. Amongst these patients, 31% had recorded pain instances in both records. While this overlap between primary and secondary care seems low, it is important to bear in mind that Lambeth only represents

For peer review only

22% of the catchment covered by CRIS [56]. Patients present in CRIS but not in LDN could include patients who have recorded instances of pain within the free-text clinical notes in LDN and might have been missed in this study since we do not have access to this text. Furthermore, this study did not differentiate between acute and chronic pain mentions and focused on extracting mentions of physical pain of any duration. As a result, the higher occurrence of pain mentioned within CRIS can be partially attributed to the documentation of such acute or short-lived pain episodes. Conversely, the GP records within LDN likely focus on recording persistent and chronic pain experiences. This disparity in recording pain should be considered when interpreting the findings of this study. Looking specifically at chronic pain instances within the CRIS notes may improve the comparability. However, the temporal information required to determine pain chronicity from clinical notes is a particular challenge and can be difficult to extract reliably. Future work can attempt to differentiate acute and chronic pain through temporal or contextual information, which could provide richer insight. However, the current broad inclusion of pain provides wider coverage for this initial exploration of pain mentioned within clinical notes.

The findings of this study highlight important considerations that need to be made regarding the assessment and management of pain among people with SMI. Existing literature demonstrates that individuals with SMI often underreport pain symptoms yet experience disproportionately high rates of chronic pain conditions compared to the general population [57,58]. The low documentation of pain in the mental health records of this cohort indicates potential gaps in detection that warrant attention, particularly given research showing links between untreated pain and worse mental health outcomes. The challenges faced by this group in communicating their pain may inhibit pain identification [59]. Additional training focused on regular, thorough pain assessment within this group is needed for mental health professionals. Specifically, potentially implementing structured screening protocols, allowing patient self-report through diverse modalities, and increased awareness of typical presentations could improve documentation and care standards. Pain assessment should become a routine aspect of comprehensive care for those with SMI to reduce compounding health decline. These steps toward more patient-centred pain management align with calls to better integrate physical and mental health services for this vulnerable population.

A strength of this study is the size of the data set available and the access to information about pain from the clinical text. To the best of our knowledge, this is potentially the first cross-sectional study to summarise and describe the distribution of recorded pain derived from routine mental health records. While the cohort data extraction did not apply any filters on demographics, aiming for broad representativeness, other systemic biases related to access to healthcare resources may still exist. Factors like deprivation level and ethnicity can influence the utilisation of services and, therefore, documentation within health records, often stemming from perceived barriers to access. However, by not restricting cohort selection on demographic factors, this study intended to capture a diverse patient population receiving care across the South London boroughs.

A limitation of this study is its reliance on clinician documentation of pain within the clinical notes, which may be subject to a form of reporting bias. Specifically, the absence of documented pain could either be because patients were asked about their pain and this information wasn't recorded, or because the patients were never asked about their pain. Absence of recorded pain does not indicate that the patients were not experiencing pain or

For peer review only

that clinicians did not inquire about pain. This study methodology does not distinguish between these scenarios. The actual occurrences of pain experiences could remain unaccounted for if they weren't recorded by the clinicians or were not shared with the clinicians, especially for patients with severe mental illnesses who might be completely or partially nonverbal. While the NLP application achieved good performance metrics during its development and evaluation [39], it is not impervious to imperfections. Instances of pain experiences might have been overlooked if they were not included as examples during the training of the application.

The scope of this study is limited to the examination of mental health records from an EHR database in South London. It is essential to acknowledge the potential influence of gender and ethnicity on the reporting of pain experiences, particularly if females and minority ethnicities (due to language barriers or other reasons) are less likely to self-report their pain experiences [55,60,61]. Since the focus of this study has been on a mental health EHR database, the clinical care within this setting is focused on mental health issues reported by the patients. Consequently, as much importance might not be given to the investigation and reporting of physical health conditions such as pain.

Conclusion

The outcomes of this study have significant implications for the assessment and management of pain amongst patients with mental health disorders and highlight the importance of utilising NLP methods on EHR databases for research purposes. Notably, these findings reiterate the recommendations set forth by Mental Health America [62], advocating the need for proactive initiation of conversations around mental health and pain with patients. Relying solely on patients to self-report symptoms could potentially lead to worse outcomes, especially since the stigma surrounding persistent pain and mental health conditions may prevent patients from seeking the necessary treatment. Thus, early and proactive interventions could go a long way towards improved long-term outcomes. Unfortunately, there still exists a perceived lack of credibility and empathy towards patients living with pain [63], particularly when compounded by co-existent mental illnesses. This was one of the main points shared by the PPI group consulted as part of this study. More research in this area can help towards these issues and provide safer and equitable access to good-quality pain management.

It is possible that some patients within the cohort, and in general within the CRIS database, might be receiving psychological therapies for persistent pain. Future research leveraging the LDN-CRIS data linkage could examine referral patterns to these services. Examining referral trends over time and across demographic factors may uncover important insights regarding access barriers, and ultimately enhance delivery of appropriate psychological care for those suffering from pain. Analysing such patterns of psychological therapy referrals using the LDN-CRIS linked data can expand our understanding of this dimension of care.

While these findings represent a step forward, they are only one side of the story. Combining these findings with patient-reported insights could offer a more comprehensive understanding of pain experiences within this cohort. However, achieving this is a challenging task due to the lack of such data and the inability to link patient-reported experiences to their health records. Further research is needed to better understand the relationship between pain and mental

For peer review only

health in this population. This could be achieved by accessing longitudinal data within this database, and studying the temporal aspects of both conditions.

Author Contributions

The idea was conceived by JC, AR, and RS. JC conducted the analyses and drafted the manuscript. MA provided insights on LDN data. AR and RS provided guidance in the design and interpretation of results. All authors commented on drafts of the manuscript and approved the final version.

Competing Interests

This paper represents independent research part-funded by the National Institute for Health Research (NIHR) Biomedical Research Centre at South London and Maudsley NHS Foundation Trust and King's College London. The views expressed are those of the authors and not necessarily those of the NHS, the NIHR or the Department of Health and Social Care. The funders had no role in study design, data collection and analysis, decision to publish, or preparation of the manuscript.

Funding

AR is funded by Health Data Research UK, an initiative funded by UK Research and Innovation, Department of Health and Social Care (England) and the devolved administrations, and leading medical research charities. RS is part-funded by i) the National Institute for Health Research (NIHR) Biomedical Research Centre at South London and Maudsley NHS Foundation Trust and King's College London; ii) the National Institute for Health Research (NIHR) Applied Research Collaboration South London (NIHR ARC South London) at King's College Hospital NHS Foundation Trust; iii) the DATAMIND HDR UK Mental Health Data Hub (MRC grant MR/W014386); iv) the UK Prevention Research Partnership (Violence, Health and Society; MR-VO49879/1), an initiative funded by UK Research and Innovation Councils, the Department of Health and Social Care (England) and the UK devolved administrations, and leading health research charities. JC is supported by the KCL-funded Centre for Doctoral Training (CDT) in Data-Driven Health. The funders were not involved in the study design, collection, analysis, interpretation of data, the writing of this article or the decision to submit it for publication. RS declared research support received in the last 36 months from Janssen, GSK and Takeda. All other authors declare no other competing interests.

This work uses data provided by patients and collected by the NHS as part of their care and support. An application for access to the Clinical Record Interactive Search (CRIS) database for this project was submitted and approved by the CRIS Oversight Committee. The authors would like to acknowledge Dr Ruimin Ma for her help in obtaining the LDN codes.

For peer review only

Data Sharing Statement

Data are owned by a third party, Maudsley Biomedical Research Centre (BRC) Clinical Records Interactive Search (CRIS) tool, which provides access to anonymised data derived from SLaM electronic medical records. These data can be accessed by permitted individuals from within a secure firewall (i.e. the data cannot be sent elsewhere) in the same manner as the authors. For more information, please contact cris.administrator@slam.nhs.uk. Any STATA and Python code used in this project will be available on GitHub[3].

Ethics Approval Statement

CRIS (as well as its associated linkages) has received ethical approval as a data resource for secondary analysis from the Oxford C Research Ethics Committee (reference 23/SC/0257). A patient-led oversight committee (detailed in [39]) reviews and approves research projects that use the CRIS database. For service users, an opt-out system is in place and is advertised in all promotional materials and campaigns. Only authorised individuals can access this data from within a secure firewall. The CRIS project approval references for this work are 21-021 and 23-003.

LDN approval was obtained as part of an existing CRIS project (project number 23-124) which included access to linked data from LDN (Caldicott Guardian approval, 15/9/21). This CRIS-LDN project aimed to examine the profile of patients with mental illnesses and chronic/persistent pain and compare them to controls from LDN who had chronic/persistent pain only.

Bibliography

1 Phillips CJ. The cost and burden of chronic pain. *Rev Pain* 2009;**3**:2–5. doi:10.1177/204946370900300102

2 Enright A, Goucke R. The global burden of pain: the tip of the iceberg? *Anesth Analg* 2016;**123**:529–30. doi:10.1213/ANE.0000000000001519

3 Merlin JS, Zinski A, Norton WE, *et al.* A conceptual framework for understanding chronic pain in patients with HIV. *Pain Pract* 2014;**14**:207–16. doi:10.1111/papr.12052

4 Meints SM, Edwards RR. Evaluating psychosocial contributions to chronic pain outcomes. *Prog Neuropsychopharmacol Biol Psychiatry* 2018;**87**:168–82. doi:10.1016/j.pnpbp.2018.01.017

5 Bridges S. Chapter 2: Mental health problem. In: *Health Survey*. 2014.

6 Abplanalp SJ, Mueser KT, Fulford D. The role of physical pain in global functioning of people with serious mental illness. *Schizophr Res* 2020;**222**:423–8. doi:10.1016/j.schres.2020.03.062

7 Public Health England. Severe mental illness (SMI) and physical health inequalities:

[3] https://github.com/jayachaturvedi/pain_in_mental_health

briefing. Research and analysis. 2018.https://www.gov.uk/government/publications/severe-mental-illness-smi-physical-health-inequalities/severe-mental-illness-and-physical-health-inequalities-briefing#:~:text=The%20phrase%20severe%20mental%20illness,an%20SMI%20%5Bfootnote%201%5D. (accessed 23 Jun2023).

8 Stubbs B, Mitchell AJ, De Hert M, *et al.* The prevalence and moderators of clinical pain in people with schizophrenia: a systematic review and large scale meta-analysis. *Schizophr Res* 2014;**160**:1–8. doi:10.1016/j.schres.2014.10.017

9 Bonnot O, Anderson GM, Cohen D, *et al.* Are patients with schizophrenia insensitive to pain? A reconsideration of the question. *Clin J Pain* 2009;**25**:244–52. doi:10.1097/AJP.0b013e318192be97

10 Birgenheir DG, Ilgen MA, Bohnert ASB, *et al.* Pain conditions among veterans with schizophrenia or bipolar disorder. *Gen Hosp Psychiatry* 2013;**35**:480–4. doi:10.1016/j.genhosppsych.2013.03.019

11 Potvin S, Marchand S. Hypoalgesia in schizophrenia is independent of antipsychotic drugs: a systematic quantitative review of experimental studies. *Pain* 2008;**138**:70–8. doi:10.1016/j.pain.2007.11.007

12 IsHak WW, Wen RY, Naghdechi L, *et al.* Pain and depression: A systematic review. *Harv Rev Psychiatry* 2018;**26**:352–63. doi:10.1097/HRP.0000000000000198

13 Bair MJ, Robinson RL, Katon W, *et al.* Depression and pain comorbidity: a literature review. *Arch Intern Med* 2003;**163**:2433–45. doi:10.1001/archinte.163.20.2433

14 Blumer D, Heilbronn M. Chronic pain as a variant of depressive disease: the pain-prone disorder. *J Nerv Ment Dis* 1982;**170**:381–406. doi:10.1097/00005053-198207000-00001

15 Rayner L, Hotopf M, Petkova H, *et al.* Depression in patients with chronic pain attending a specialised pain treatment centre: prevalence and impact on health care costs. *Pain* 2016;**157**:1472–9. doi:10.1097/j.pain.0000000000000542

16 Thompson T, Correll CU, Gallop K, *et al.* Is Pain Perception Altered in People With Depression? A Systematic Review and Meta-Analysis of Experimental Pain Research. *J Pain* 2016;**17**:1257–72. doi:10.1016/j.jpain.2016.08.007

17 Noroozian M, Raeesi S, Hashemi R, *et al.* Pain: the neglect issue in old people's life. *Open Access Maced J Med Sci* 2018;**6**:1773–8. doi:10.3889/oamjms.2018.335

18 Fillingim RB. Sex, gender, and pain. In: *Principles of Gender-Specific Medicine*. Elsevier 2017. 481–96. doi:10.1016/B978-0-12-803506-1.00038-3

19 Vallerand AH, Polomano RC. The relationship of gender to pain. *Pain Manag Nurs* 2000;**1**:8–15. doi:10.1053/jpmn.2000.9759

20 Campbell CM, Edwards RR. Ethnic differences in pain and pain management. *Pain Manag* 2012;**2**:219–30. doi:10.2217/pmt.12.7

21 Martin JL, McLean G, Park J, *et al.* Impact of socioeconomic deprivation on rate and cause of death in severe mental illness. *BMC Psychiatry* 2014;**14**:261. doi:10.1186/s12888-014-0261-4

22 Lambert TJR, Newcomer JW. Are the cardiometabolic complications of schizophrenia still neglected? Barriers to care. *Med J Aust* 2009;**190**:S39-42. doi:10.5694/j.1326-

For peer review only

5377.2009.tb02374.x

23 DE Hert M, Correll CU, Bobes J, *et al.* Physical illness in patients with severe mental disorders. I. Prevalence, impact of medications and disparities in health care. *World Psychiatry* 2011;**10**:52–77. doi:10.1002/j.2051-5545.2011.tb00014.x

24 Frayne SM, Halanych JH, Miller DR, *et al.* Disparities in diabetes care: impact of mental illness. *Arch Intern Med* 2005;**165**:2631–8. doi:10.1001/archinte.165.22.2631

25 Laursen TM, Munk-Olsen T, Agerbo E, *et al.* Somatic hospital contacts, invasive cardiac procedures, and mortality from heart disease in patients with severe mental disorder. *Arch Gen Psychiatry* 2009;**66**:713–20. doi:10.1001/archgenpsychiatry.2009.61

26 Aro S, Aro H, Keskimäki I. Socio-economic mobility among patients with schizophrenia or major affective disorder. A 17-year retrospective follow-up. *Br J Psychiatry* 1995;**166**:759–67. doi:10.1192/bjp.166.6.759

27 Barnett P, Oshinowo I, Cooper C, *et al.* The association between social class and the impact of treatment for mental health problems: a systematic review and narrative synthesis. *Soc Psychiatry Psychiatr Epidemiol* 2023;**58**:581–603. doi:10.1007/s00127-022-02378-9

28 Lawrence D, Kisely S. Inequalities in healthcare provision for people with severe mental illness. *J Psychopharmacol (Oxford)* 2010;**24**:61–8. doi:10.1177/1359786810382058

29 Velupillai S, Suominen H, Liakata M, *et al.* Using clinical Natural Language Processing for health outcomes research: Overview and actionable suggestions for future advances. *J Biomed Inform* 2018;**88**:11–9. doi:10.1016/j.jbi.2018.10.005

30 Kadra G, Stewart R, Shetty H, *et al.* Long-term antipsychotic polypharmacy prescribing in secondary mental health care and the risk of mortality. *Acta Psychiatr Scand* 2018;**138**:123–32. doi:10.1111/acps.12906

31 Bendayan R, Kraljevic Z, Shaari S, *et al.* Mapping multimorbidity in individuals with schizophrenia and bipolar disorders: evidence from the South London and Maudsley NHS Foundation Trust Biomedical Research Centre (SLAM BRC) case register. *BMJ Open* 2022;**12**:e054414. doi:10.1136/bmjopen-2021-054414

32 Jackson RG, Patel R, Jayatilleke N, *et al.* Natural language processing to extract symptoms of severe mental illness from clinical text: the Clinical Record Interactive Search Comprehensive Data Extraction (CRIS-CODE) project. *BMJ Open* 2017;**7**:e012012. doi:10.1136/bmjopen-2016-012012

33 Sampson R, Cooper J, Barbour R, *et al.* Patients' perspectives on the medical primary-secondary care interface: systematic review and synthesis of qualitative research. *BMJ Open* 2015;**5**:e008708. doi:10.1136/bmjopen-2015-008708

34 Benchimol EI, Smeeth L, Guttmann A, *et al.* [The REporting of studies Conducted using Observational Routinely-collected health Data (RECORD) statement]. *Z Evid Fortbild Qual Gesundhwes* 2016;**115–116**:33–48. doi:10.1016/j.zefq.2016.07.010

35 von Elm E, Altman DG, Egger M, *et al.* Strengthening the Reporting of Observational Studies in Epidemiology (STROBE) statement: guidelines for reporting observational studies. *BMJ* 2007;**335**:806–8. doi:10.1136/bmj.39335.541782.AD

36 Stewart R, Soremekun M, Perera G, *et al.* The South London and Maudsley NHS

For peer review only

Foundation Trust Biomedical Research Centre (SLAM BRC) case register: development and descriptive data. *BMC Psychiatry* 2009;**9**:51. doi:10.1186/1471-244X-9-51

37 N H S. Lambeth DataNet. Lambeth DataNet. 2021.https://selondonccg.nhs.uk/wp-content/uploads/2021/04/Lambeth-DataNet.pdf (accessed 26 May2023).

38 NIHR Maudsley Biomedical Research Center. CRIS Data Linkages. CRIS Data Linkages. https://www.maudsleybrc.nihr.ac.uk/facilities/clinical-record-interactive-search-cris/cris-data-linkages/ (accessed 7 Aug2023).

39 Fernandes AC, Cloete D, Broadbent MTM, *et al.* Development and evaluation of a de-identification procedure for a case register sourced from mental health electronic records. *BMC Med Inform Decis Mak* 2013;**13**:71. doi:10.1186/1472-6947-13-71

40 Research Design Service South Central. Patient and Public Involvement (PPI). What is Patient and Public Involvement in health and social care research? 2023.https://www.rds-sc.nihr.ac.uk/ppi-information-resources/

41 World Health Organization. International Classification of Diseases (ICD). 2008.https://www.who.int/standards/classifications/classification-of-diseases (accessed 7 Aug2023).

42 GOV.UK. English indices of deprivation 2019. 2019.https://www.gov.uk/government/statistics/english-indices-of-deprivation-2019 (accessed 7 Aug2023).

43 Chaturvedi J, Mascio A, Velupillai SU, *et al.* Development of a lexicon for pain. *Front Digit Health* 2021;**3**:778305. doi:10.3389/fdgth.2021.778305

44 Liu F, Shareghi E, Meng Z, *et al.* Self-Alignment Pretraining for Biomedical Entity Representations. *arXiv:201011784 [cs]* 2021.

45 Chaturvedi J, Velupillai S, Stewart R, et al. Identifying mentions of pain in mental health records text: A natural language processing approach. Stud Health Technol Inform 2024;310:695–9. doi:10.3233/SHTI231054

46 NHS Digital. Read Codes. 2022.https://digital.nhs.uk/services/terminology-and-classifications/read-codes (accessed 7 Aug2023).

47 Ma R, Romano E, Ashworth M, *et al.* Multimorbidity clusters among people with serious mental illness: a representative primary and secondary data linkage cohort study. *Psychol Med* 2022;:1–12. doi:10.1017/S003329172200109X

48 NHSBSA. Dictionary of medicines and devices (dm+d) . 2023.https://www.nhsbsa.nhs.uk/pharmacies-gp-practices-and-appliance-contractors/dictionary-medicines-and-devices-dmd (accessed 2 Aug2023).

49 Barnett K, Mercer SW, Norbury M, *et al.* Epidemiology of multimorbidity and implications for health care, research, and medical education: a cross-sectional study. *Lancet* 2012;**380**:37–43. doi:10.1016/S0140-6736(12)60240-2

50 Outcomes Based Healthcare. Outcomes Platform. 2018.https://outcomesbasedhealthcare.com/outcomes-platform/ (accessed 3 Aug2023).

51 Hafezparast N, Bragan Turner E, Dunbar-Rees R, *et al.* Identifying populations with chronic pain in primary care: developing an algorithm and logic rules applied to coded primary care diagnostic and medication data. *BMC Primary Care* 2023.

For peer review only

52 Searle T, Kraljevic Z, Bendayan R, *et al.* MedCATTrainer: A Biomedical Free Text Annotation Interface with Active Learning and Research Use Case Specific Customisation. In: *Proceedings of the 2019 Conference on Empirical Methods in Natural Language Processing and the 9th International Joint Conference on Natural Language Processing (EMNLP-IJCNLP): System Demonstrations*. Stroudsburg, PA, USA: : Association for Computational Linguistics 2019. 139–44. doi:10.18653/v1/D19-3024

53 Wandner LD, Scipio CD, Hirsh AT, *et al.* The perception of pain in others: how gender, race, and age influence pain expectations. *J Pain* 2012;**13**:220–7. doi:10.1016/j.jpain.2011.10.014

54 Fillingim RB. Sex, gender, and pain: women and men really are different. *Curr Rev Pain* 2000;**4**:24–30. doi:10.1007/s11916-000-0006-6

55 Green CR, Anderson KO, Baker TA, *et al.* The unequal burden of pain: confronting racial and ethnic disparities in pain. *Pain Med* 2003;**4**:277–94. doi:10.1046/j.1526-4637.2003.03034.x

56 Perera G, Broadbent M, Callard F, *et al.* Cohort profile of the South London and Maudsley NHS Foundation Trust Biomedical Research Centre (SLaM BRC) Case Register: current status and recent enhancement of an Electronic Mental Health Record-derived data resource. *BMJ Open* 2016;**6**:e008721. doi:10.1136/bmjopen-2015-008721

57 Brooks JM, Polenick CA, Bryson W, *et al.* Pain intensity, depressive symptoms, and functional limitations among older adults with serious mental illness. *Aging Ment Health* 2019;**23**:470–4. doi:10.1080/13607863.2017.1423025

58 Engels G, Francke AL, van Meijel B, *et al.* Clinical pain in schizophrenia: a systematic review. *J Pain* 2014;**15**:457–67. doi:10.1016/j.jpain.2013.11.005

59 Onwumere J, Stubbs B, Stirling M, *et al.* Pain management in people with severe mental illness: an agenda for progress. *Pain* 2022;**163**:1653–60. doi:10.1097/j.pain.0000000000002633

60 Samulowitz A, Gremyr I, Eriksson E, *et al.* “Brave Men” and “Emotional Women”: A Theory-Guided Literature Review on Gender Bias in Health Care and Gendered Norms towards Patients with Chronic Pain. *Pain Res Manag* 2018;**2018**:6358624. doi:10.1155/2018/6358624

61 Hoffmann DE, Tarzian AJ. The girl who cried pain: a bias against women in the treatment of pain. *J Law Med Ethics* 2001;**29**:13–27. doi:10.1111/j.1748-720X.2001.tb00037.x

62 American Psychiatric Association. Chronic Pain and Mental Health Often Interconnected. 2020.https://www.psychiatry.org/news-room/apa-blogs/chronic-pain-and-mental-health-interconnected (accessed 3 Aug2023).

63 Bennett C. What you need to know about pain and mental health in Australia. Pain Australia. 2020.https://www.painaustralia.org.au/media-document/blog-1/blog-2020/blog-2019/what-you-need-to-know-about-pain-and-mental-health-in-australia (accessed 3 Aug2023).

For peer review only

Figure Legend

Figure 1 – Data Extraction
Figure 2 – Overlap of recorded pain in CRIS and LDN

For peer review only

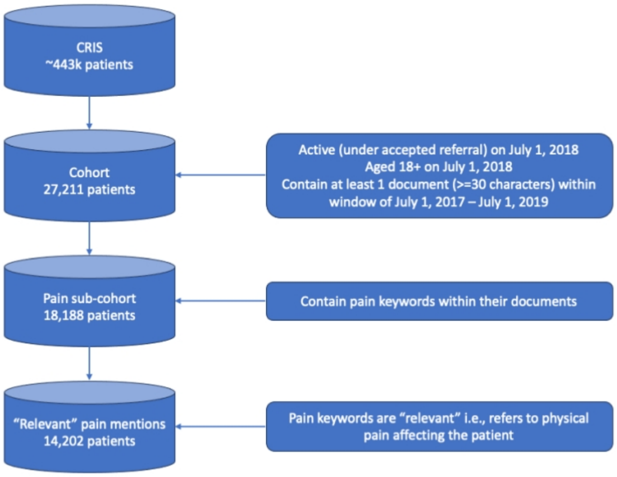

Figure 1. Data Extraction

339x191mm (102 x 102 DPI)

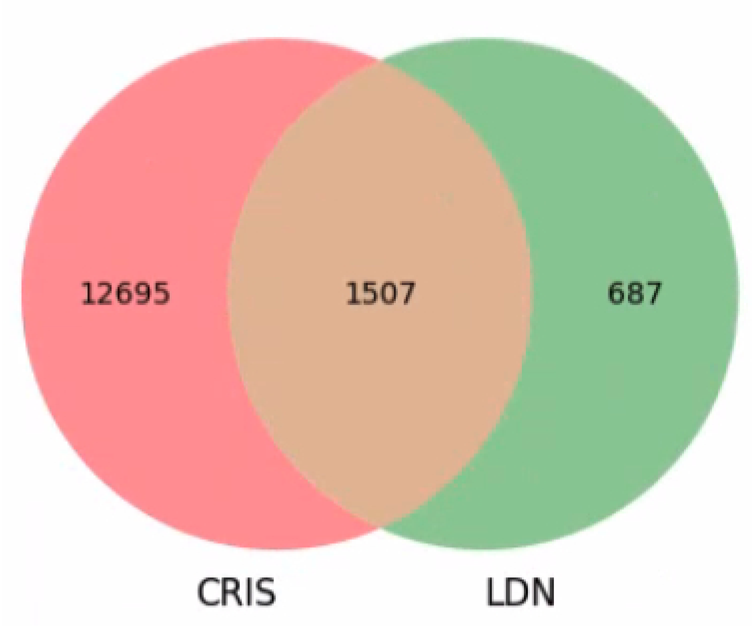

Figure 2. Overlap of recorded pain in CRIS and LDN

132x116mm (72 x 72 DPI)

Reporting checklist for cross sectional study.

Based on the STROBE cross sectional guidelines.

Instructions to authors

Complete this checklist by entering the page numbers from your manuscript where readers will find each of the items listed below.

Your article may not currently address all the items on the checklist. Please modify your text to include the missing information. If you are certain that an item does not apply, please write "n/a" and provide a short explanation.

Upload your completed checklist as an extra file when you submit to a journal.

In your methods section, say that you used the STROBE cross sectionalreporting guidelines, and cite them as:

von Elm E, Altman DG, Egger M, Pocock SJ, Gotzsche PC, Vandenbroucke JP. The Strengthening the Reporting of Observational Studies in Epidemiology (STROBE) Statement: guidelines for reporting observational studies.

		Reporting Item	Page Number
**Title and abstract**			
Title	#1a	Indicate the study's design with a commonly used term in the title or the abstract	1
Abstract	#1b	Provide in the abstract an informative and balanced summary of what was done and what was found	1
**Introduction**			
Background / rationale	#2	Explain the scientific background and rationale for the investigation being reported	2
Objectives	#3	State specific objectives, including any prespecified hypotheses	4
**Methods**			
Study design	#4	Present key elements of study design early in the paper	4
Setting	#5	Describe the setting, locations, and relevant dates, including periods of	4

For peer review only

		recruitment, exposure, follow-up, and data collection	
Eligibility criteria	#6a	Give the eligibility criteria, and the sources and methods of selection of participants.	5
	#7	Clearly define all outcomes, exposures, predictors, potential confounders, and effect modifiers. Give diagnostic criteria, if applicable	5
Data sources / measurement	#8	For each variable of interest give sources of data and details of methods of assessment (measurement). Describe comparability of assessment methods if there is more than one group. Give information separately for for exposed and unexposed groups if applicable.	5-7
Bias	#9	Describe any efforts to address potential sources of bias	14
Study size	#10	Explain how the study size was arrived at	5
Quantitative variables	#11	Explain how quantitative variables were handled in the analyses. If applicable, describe which groupings were chosen, and why	6
Statistical methods	#12a	Describe all statistical methods, including those used to control for confounding	8
Statistical methods	#12b	Describe any methods used to examine subgroups and interactions	8
Statistical methods	#12c	Explain how missing data were addressed	6
Statistical methods	#12d	If applicable, describe analytical methods taking account of sampling strategy	n/a
Statistical methods	#12e	Describe any sensitivity analyses	n/a
**Results**			
Participants	#13a	Report numbers of individuals at each stage of study—eg numbers potentially eligible, examined for eligibility, confirmed eligible, included in the study, completing follow-up, and analysed. Give information separately for for exposed and unexposed groups if applicable.	8
Participants	#13b	Give reasons for non-participation at each stage	8
Participants	#13c	Consider use of a flow diagram	8

For peer review only

Descriptive data	#14a	Give characteristics of study participants (eg demographic, clinical, social) and information on exposures and potential confounders. Give information separately for exposed and unexposed groups if applicable.	8-9
Descriptive data	#14b	Indicate number of participants with missing data for each variable of interest	9
Outcome data	#15	Report numbers of outcome events or summary measures. Give information separately for exposed and unexposed groups if applicable.	9-10
Main results	#16a	Give unadjusted estimates and, if applicable, confounder-adjusted estimates and their precision (eg, 95% confidence interval). Make clear which confounders were adjusted for and why they were included	10-11
Main results	#16b	Report category boundaries when continuous variables were categorized	11
Main results	#16c	If relevant, consider translating estimates of relative risk into absolute risk for a meaningful time period	n/a
Other analyses	#17	Report other analyses done—e.g., analyses of subgroups and interactions, and sensitivity analyses	12-13
**Discussion**			
Key results	#18	Summarise key results with reference to study objectives	13
Limitations	#19	Discuss limitations of the study, taking into account sources of potential bias or imprecision. Discuss both direction and magnitude of any potential bias.	13-15
Interpretation	#20	Give a cautious overall interpretation considering objectives, limitations, multiplicity of analyses, results from similar studies, and other relevant evidence.	13-15
Generalisability	#21	Discuss the generalisability (external validity) of the study results	13-15
**Other Information**			
Funding	#22	Give the source of funding and the role of the funders for the present study and, if applicable, for the original study on which the present article is based	16

The STROBE checklist is distributed under the terms of the Creative Commons Attribution License CC-BY. This checklist was completed on 24. August 2023 using https://www.goodreports.org/, a tool made by the EQUATOR Network in collaboration with Penelope.ai

For peer review only

The RECORD statement – checklist of items, extended from the STROBE statement, that should be reported in observational studies using routinely collected health data.

	Item No.	STROBE items	Location in manuscript where items are reported	RECORD items	Location in manuscript where items are reported
**Title and abstract**					
	1	(a) Indicate the study's design with a commonly used term in the title or the abstract (b) Provide in the abstract an informative and balanced summary of what was done and what was found	(a) Title<br>(b) Abstract	RECORD 1.1: The type of data used should be specified in the title or abstract. When possible, the name of the databases used should be included.<br><br>RECORD 1.2: If applicable, the geographic region and timeframe within which the study took place should be reported in the title or abstract.<br><br>RECORD 1.3: If linkage between databases was conducted for the study, this should be clearly stated in the title or abstract.	Abstract
**Introduction**					
Background rationale	2	Explain the scientific background and rationale for the investigation being reported	Introduction - Background Rationale		
Objectives	3	State specific objectives, including any prespecified hypotheses	Introduction - Objectives		
**Methods**					
Study Design	4	Present key elements of study design early in the paper	Methods		
Setting	5	Describe the setting, locations, and relevant dates, including periods of recruitment, exposure, follow-up, and data collection	Methods - Setting and Variables		

For peer review only

Participants	6	*(a) Cohort study* - Give the eligibility criteria, and the sources and methods of selection of participants. Describe methods of follow-up<br>*Case-control study* - Give the eligibility criteria, and the sources and methods of case ascertainment and control selection. Give the rationale for the choice of cases and controls<br>*Cross-sectional study* - Give the eligibility criteria, and the sources and methods of selection of participants<br><br>*(b) Cohort study* - For matched studies, give matching criteria and number of exposed and unexposed<br>*Case-control study* - For matched studies, give matching criteria and the number of controls per case	Methods - Participants	RECORD 6.1: The methods of study population selection (such as codes or algorithms used to identify subjects) should be listed in detail. If this is not possible, an explanation should be provided.<br><br>RECORD 6.2: Any validation studies of the codes or algorithms used to select the population should be referenced. If validation was conducted for this study and not published elsewhere, detailed methods and results should be provided.<br><br>RECORD 6.3: If the study involved linkage of databases, consider use of a flow diagram or other graphical display to demonstrate the data linkage process, including the number of individuals with linked data at each stage.	
Variables	7	Clearly define all outcomes, exposures, predictors, potential confounders, and effect modifiers. Give diagnostic criteria, if applicable.	Methods - Variables	RECORD 7.1: A complete list of codes and algorithms used to classify exposures, outcomes, confounders, and effect modifiers should be provided. If these cannot be reported, an explanation should be provided.	
Data sources/ measurement	8	For each variable of interest, give sources of data and details of methods of assessment (measurement).<br>Describe comparability of assessment methods if there is more than one group	Methods - Variables		

For peer review only

Bias	9	Describe any efforts to address potential sources of bias	Methods - Variables		
Study size	10	Explain how the study size was arrived at	Methods - Participants		
Quantitative variables	11	Explain how quantitative variables were handled in the analyses. If applicable, describe which groupings were chosen, and why	Methods - Variables		
Statistical methods	12	(a) Describe all statistical methods, including those used to control for confounding<br>(b) Describe any methods used to examine subgroups and interactions<br>(c) Explain how missing data were addressed<br>(d) *Cohort study* - If applicable, explain how loss to follow-up was addressed<br>*Case-control study* - If applicable, explain how matching of cases and controls was addressed<br>*Cross-sectional study* - If applicable, describe analytical methods taking account of sampling strategy<br>(e) Describe any sensitivity analyses	Methods - Setting and Descriptive Statistics		
Data access and cleaning methods		..		RECORD 12.1: Authors should describe the extent to which the investigators had access to the database population used to create the study population.	Methods - Setting - Ethical Approval

For peer review only

				RECORD 12.2: Authors should provide information on the data cleaning methods used in the study.	Methods - Variables
Linkage		..		RECORD 12.3: State whether the study included person-level, institutional-level, or other data linkage across two or more databases. The methods of linkage and methods of linkage quality evaluation should be provided.	Methods - Setting
**Results**					
Participants	13	(a) Report the numbers of individuals at each stage of the study (*e.g.*, numbers potentially eligible, examined for eligibility, confirmed eligible, included in the study, completing follow-up, and analysed)<br>(b) Give reasons for non-participation at each stage.<br>(c) Consider use of a flow diagram	Results	RECORD 13.1: Describe in detail the selection of the persons included in the study (*i.e.*, study population selection) including filtering based on data quality, data availability and linkage. The selection of included persons can be described in the text and/or by means of the study flow diagram.	Methods
Descriptive data	14	(a) Give characteristics of study participants (*e.g.*, demographic, clinical, social) and information on exposures and potential confounders<br>(b) Indicate the number of participants with missing data for each variable of interest<br>(c) *Cohort study* - summarise follow-up time (*e.g.*, average and total amount)	Results		
Outcome data	15	*Cohort study* - Report numbers of outcome events or summary measures over time<br>*Case-control study* - Report numbers in each exposure	Results		

For peer review only

		category, or summary measures of exposure<br>*Cross-sectional study* - Report numbers of outcome events or summary measures			
Main results	16	(a) Give unadjusted estimates and, if applicable, confounder-adjusted estimates and their precision (e.g., 95% confidence interval). Make clear which confounders were adjusted for and why they were included<br>(b) Report category boundaries when continuous variables were categorized<br>(c) If relevant, consider translating estimates of relative risk into absolute risk for a meaningful time period	Results		
Other analyses	17	Report other analyses done—e.g., analyses of subgroups and interactions, and sensitivity analyses	Results		
**Discussion**					
Key results	18	Summarise key results with reference to study objectives	Discussion		
Limitations	19	Discuss limitations of the study, taking into account sources of potential bias or imprecision. Discuss both direction and magnitude of any potential bias	Discussion	RECORD 19.1: Discuss the implications of using data that were not created or collected to answer the specific research question(s). Include discussion of misclassification bias, unmeasured confounding, missing data, and changing eligibility over time, as they pertain to the study being reported.	Discussion and Conclusion
Interpretation	20	Give a cautious overall interpretation of results considering objectives,			

For peer review only

		limitations, multiplicity of analyses, results from similar studies, and other relevant evidence	Discussion		
Generalisability	21	Discuss the generalisability (external validity) of the study results	Discussion		
**Other Information**					
Funding	22	Give the source of funding and the role of the funders for the present study and, if applicable, for the original study on which the present article is based	Acknolwledgements and disclosure of interests		
Accessibility of protocol, raw data, and programming code		..	Data Availability Statement	RECORD 22.1: Authors should provide information on how to access any supplemental information such as the study protocol, raw data, or programming code.	Data Availability Statement

*Reference: Benchimol EI, Smeeth L, Guttmann A, Harron K, Moher D, Petersen I, Sørensen HT, von Elm E, Langan SM, the RECORD Working Committee. The REporting of studies Conducted using Observational Routinely-collected health Data (RECORD) Statement. *PLoS Medicine* 2015; in press.

*Checklist is protected under Creative Commons Attribution (CC BY) license.

For peer review only

BMJ Open

Distributions of Recorded Pain in Mental Health Records: A Natural Language Processing Based Study

Journal:	*BMJ Open*
Manuscript ID	bmjopen-2023-079923.R2
Article Type:	Original research
Date Submitted by the Author:	27-Feb-2024
Complete List of Authors:	Chaturvedi, Jaya; King's College London, Department of Biostatistics and Health Informatics<br>Stewart, Robert; King's College London, Institute of Psychiatry<br>Ashworth, Mark; King's College London, School of Population Health & Environmental Sciences<br>Roberts, Angus; King's College London, Institute of Psychiatry, Psychology and Neuroscience
**Primary Subject Heading**:	Health informatics
Secondary Subject Heading:	Epidemiology, Mental health
Keywords:	Natural Language Processing, Electronic Health Records, EPIDEMIOLOGY, MENTAL HEALTH, Chronic Pain

SCHOLARONE™
Manuscripts

I, the Submitting Author has the right to grant and does grant on behalf of all authors of the Work (as defined in the below author licence), an exclusive licence and/or a non-exclusive licence for contributions from authors who are: i) UK Crown employees; ii) where BMJ has agreed a CC-BY licence shall apply, and/or iii) in accordance with the terms applicable for US Federal Government officers or employees acting as part of their official duties; on a worldwide, perpetual, irrevocable, royalty-free basis to BMJ Publishing Group Ltd ("BMJ") its licensees and where the relevant Journal is co-owned by BMJ to the co-owners of the Journal, to publish the Work in this journal and any other BMJ products and to exploit all rights, as set out in our licence.

The Submitting Author accepts and understands that any supply made under these terms is made by BMJ to the Submitting Author unless you are acting as an employee on behalf of your employer or a postgraduate student of an affiliated institution which is paying any applicable article publishing charge ("APC") for Open Access articles. Where the Submitting Author wishes to make the Work available on an Open Access basis (and intends to pay the relevant APC), the terms of reuse of such Open Access shall be governed by a Creative Commons licence – details of these licences and which Creative Commons licence will apply to this Work are set out in our licence referred to above.

Other than as permitted in any relevant BMJ Author's Self Archiving Policies, I confirm this Work has not been accepted for publication elsewhere, is not being considered for publication elsewhere and does not duplicate material already published. I confirm all authors consent to publication of this Work and authorise the granting of this licence.

For peer review only

Distributions of Recorded Pain in Mental Health Records: A Natural Language Processing Based Study

Jaya Chaturvedi[1*], Robert Stewart[1,2], Mark Ashworth[1], Angus Roberts[1]
[1]Institute of Psychiatry, Psychology and Neurosciences, King's College London
[2]South London and Maudsley NHS Foundation Trust
*Corresponding author: jaya.1.chaturvedi@kcl.ac.uk

For peer review only

Abstract

Objective

The objective of this study is to determine demographic and diagnostic distributions of physical pain recorded in clinical notes of a mental health electronic health records database by utilising natural language processing and examine the overlap in recorded physical pain between primary and secondary care.

Design, Setting and Participants

The data were extracted from an anonymised version of the electronic health records from a large secondary mental healthcare provider serving a catchment of 1.3M residents in south London. These included patients under active referral, aged 18+ at index date of July 1, 2018, and had at least one clinical document (>=30 characters) between July 1, 2017 and July 1, 2019. This cohort was compared to linked primary care records from one of the four local government areas.

Outcome

The primary outcome of interest was the presence of recorded physical pain within the clinical notes of the patients, not including psychological or metaphorical pain.

Results

A total of 27,211 patients were retrieved. Of these, 52% (14,202) had narrative text containing relevant mentions of physical pain. Older patients (OR 1.17, 95% CI 1.15-1.19), female (OR 1.42, 95% CI 1.35-1.49), Asian (OR 1.30, 95% CI 1.16-1.45) or Black (OR 1.49, 95% CI 1.40-1.59) ethnicities, living in deprived neighbourhoods (OR 1.64, 95% CI 1.55-1.73) showed higher odds of recorded pain. Patients with severe mental illnesses were found to be less likely to report pain (OR 0.43, 95% CI 0.41-0.46, p<0.001). 17% of the cohort from secondary care also had records within primary care.

Conclusion

The findings of this study show sociodemographic and diagnostic differences in recorded pain. Specifically, lower documentation across certain groups indicates needs for better screening protocols and training on recognising varied pain presentations. Additionally, targeting improved detection of pain for minority/disadvantaged groups by care providers can promote health equity.

Keywords: Natural Language Processing, Pain, Mental Health, Electronic Health Records

Word count: 4,880

Strengths and Limitations of this study

- This study utilises natural language processing on clinical notes to access a large sample with information about pain. The identification of such information would not be feasible manually
- This is the first cross-sectional study to summarise and describe the distribution of recorded pain within the clinical notes of mental health records.
- Inclusion of both secondary mental health and primary care records for the same patients allows comparison of pain documentation across different health services.
- When patients show no recorded pain, the study does not differentiate between pain that was discussed but not recorded, or pain that was not discussed.
- The findings are not generalisable to the general population since this study only looks at patients receiving mental healthcare within a specific geographic catchment.

Introduction

Background Rationale

Pain and its relationship with mental health are important research topics. Pain has imposed a significant burden on society in terms of medical care costs as well as lost productivity [1,2]. Pain is multifaceted, with physical, psychological, social, and biological causes and consequences [3,4]. Pain, in this context, refers to any pain condition or symptom, acute or chronic. Mental health disorders also present a considerable and complex public health problem, being a leading cause of disability and accounting for 28% of the national disease burden in the UK [5]. Electronic health records (EHRs) for mental health are a significant source of information for studying the intersection between pain and mental health within those who receive specialist service input. EHRs open the possibility of investigating how pain is recorded and its impact on clinical outcomes.

Severe mental illnesses (SMIs) include diagnoses of schizophrenia spectrum disorder, bipolar disorder, or severe major depressive disorder [6], where functional and occupational activities are severely impaired due to associated debilitating psychological problems [7]. While several studies have looked at the relationship between pain and schizophrenia and bipolar disorders [8–11] and at other mental illnesses such as depression [12–16], the complex and potentially

For peer review only

bidirectional nature of this relationship requires further understanding. Painful conditions occuring as physical comorbidities alongside mental health issues can exacerbate both conditions, with each impacting the management of the other. The combination of pain and depression, for instance, affects mental, physical and social functioning [12]. Furthermore, patients with schizophrenia tend to underreport their pain [9]. Analysis of secondary data sources, such as EHR databases, might help by providing a fuller picture of the recorded clinical presentation of this group of patients; however, a prerequisite is that pain is adequately represented in derived data.

Demographic features such as age, gender and ethnicity may affect how pain is perceived and experienced. Pain affects twice as many persons over the age of 60 as it does younger individuals [17]. While pain is not a natural feature of the ageing process, many health conditions causing pain become more common with increasing age. Nonetheless, older patients often believe pain to be a normal aspect of ageing and might be hesitant when reporting it [17]. There have also been variations found in the reported perception of pain by female and male patients, with female patients reporting experiencing more pain than males [18,19]. Research has also shown disparities in pain perception across different ethnicities, with individuals of Black African ethnicity reporting greater pain than White counterparts [20].

Socioeconomic status (SES) plays a role in health and overall well-being, with deprivation associated with unfavourable health outcomes and increased mortality rates [21]. Patients with SMI already experience higher mortality rates than the general population, and this discrepancy is exacerbated by socioeconomic deprivation, primarily due to unequal access to good quality physical healthcare services [22–25]. Furthermore, patients with SMI continue to experience a decline in their SES over time, compounding its impact [26]. Given these well-established connections between lower SES and reduced access to care [27,28], examination of potential SES-based differences in documentation of physical symptoms such as pain is particularly relevant. As disadvantaged patients face barriers in the screening for comorbid conditions, this may manifest in lower rates of discussions and recording of pain symptoms.

Most patient information is recorded in unstructured clinical narratives within EHR databases [29], and pain is likely to be no different, with few, if any, structured checklists ascertaining its presence in routine clinical care. Natural language processing (NLP), a computational approach to understanding and analysing human language, is therefore potentially useful for extracting such pain information. NLP has been applied extensively to EHR data, including studies of SMI, such as antipsychotic polypharmacy in mental health care [30], multimorbidity in individuals with schizophrenia and bipolar disorders [31], and extracting symptoms of SMI [32].

In addition to secondary care data, it is also useful to consider the recording of pain in primary care data. Within the UK, primary care (GP - general practice) is generally the first point of contact for patients [33]. Exploring the overlap of recorded pain between primary care GP services and secondary care mental health services could, therefore, provide a more comprehensive view of the patient's pain experiences, and any discrepancies could highlight gaps in care and communication across different parts of the healthcare system. As primary care physicians are often responsible for the initial pain assessment and referral to specialists if needed, documentation patterns in GP records versus mental health provider records may differ for those with psychiatric disorders. Comparing recorded pain rates across these care

For peer review only

settings can reveal insights into the consistency of pain screening among this vulnerable population across the healthcare landscape. This study utilised GP records specifically for patients contained within a mental health secondary care database in order to explore documentation patterns in primary care for patients who were recorded in secondary care with a mental illness. Analysis of these GP records takes the documentation beyond specialist mental health settings, and provides valuable insights into the larger healthcare experiences of those with mental health disorders. Additionally, examination of potential differences or overlap between the primary and secondary care for the same patient cohort enables important observations about consistency of pain assessment across providers.

Objectives

The objective of this study is to describe the distributions of recorded pain amongst mental health service users according to demographic factors such as age, gender and ethnicity, as well as neighbourhood deprivation levels and mental health diagnoses. This was achieved by examining recorded pain through the means of an NLP application within the clinical text of a mental health EHR database, and further evaluating this by measuring the overlap between pain recorded in secondary and primary health care, enabled through data linkage between the two.

This research aims to address knowledge gaps regarding the documentation of pain among mental health populations. In particular, a clearer understanding of these patterns is essential given the exceptionally high rates of pain conditions comorbid with mental health disorders. This study will answer fundamental questions around the frequencies of documented pain discussions during mental health encounters, consistency in pain detection across primary vs secondary care settings, and whether certain groups defined by gender, ethnicity or socioeconomic status face greater gaps in pain inquiry documentation. By analysing rates and differences of recorded pain within mental health records using a population-based cohort, this study works towards addressing needs around appropriate pain identification as a routine component of comprehensive mental health treatment.

Methods

Reporting

We use the RECORD [34] guidelines and checklist, an extension of the STROBE [35] guidelines, for reporting the results of this study.

Setting

Data on recorded pain, which in this context refers to any mentions of physical pain within the clinical notes, such as “complains of pain” and “experiencing headaches”, were obtained from the clinical text of a mental health EHR database, the Clinical Record Interactive Search (CRIS) resource. This contains a de-identified version of EHR data from The South London and Maudsley NHS Foundation Trust (SLaM), one of Europe's largest mental healthcare organisations [36], which serves a geographic catchment of around 1.3 million residents in

For peer review only

four south London boroughs (Croydon, Lambeth, Lewisham, Southwark). CRIS contains about 30 million free text documents, averaging 90 documents per patient [29].

Data were also obtained from a primary care database called Lambeth DataNet (LDN) [37], which accesses all GP records from general practices based in the London borough of Lambeth. Data linkages (at the patient level) are already in place between CRIS and LDN [38].

Ethical Approval

CRIS (as well as its associated linkages) has received ethical approval as a data resource for secondary analysis from the Oxford C Research Ethics Committee (reference 23/SC/0257). A patient-led oversight committee (detailed in [39]) reviews and approves research projects that use the CRIS database. For service users, an opt-out system is in place and is advertised in all promotional materials and campaigns. Only authorised individuals can access this data from within a secure firewall. The CRIS project approval references for this work are 21-021 and 23-003.

LDN approval was obtained as part of an existing CRIS project (project number 23-124) which included access to linked data from LDN (Caldicott Guardian approval, 15/9/21). This CRIS-LDN project aimed to examine the profile of patients with mental illnesses and chronic/persistent pain and compare them to controls from LDN who had chronic/persistent pain only.

Patient and Public Involvement

Patient and public involvement (PPI) in research is an active collaboration between researchers and members of the public, where the latter actively participate in contributing to the research [40]. A PPI group with lived experiences of SMI and chronic pain were consulted as part of this research. The nature of the data available was described to the group, and they were asked about their priorities regarding what research questions they would like answered. In response to this, the group was unanimously interested in further study of the differences in pain experiences based on demographics and diagnoses, and this was the main motivation for the objective of the study described here.

Participants

A cohort of patients was extracted from the CRIS database comprising those who were active (i.e., the secondary care hospital trust (SLaM) has accepted them as a referral) and aged 18+ on the index date of July 1, 2018, and whose record contained at least one document (>= 30 characters) within a window of July 1, 2017 to July 1, 2019.

LDN extraction followed similar criteria for patients who were active on the index date, aged 18+, and contained pain diagnoses or medications from July 1, 2017 to July 1, 2019. Free-text information is unavailable within LDN, so no document criteria were required.

For peer review only

Variables

Demographics

Age, gender, and ethnicity variables were extracted from structured tables within the CRIS database. Individuals with missing ethnicity values were retained as a separate category (Not stated/known). Ethnicity, in this context, encompasses both race and ethnicity but is referred to simply as ethnicity for the sake of simplicity.

Diagnosis

The primary diagnosis recorded closest to the index date of July 1, 2018, was extracted from the structured tables within the CRIS database. These are coded using ICD-10 [41]. The diagnosis codes were categorised as SMI and non-SMI, where SMI includes ICD-10 codes of F20-29 and F30-33.

Deprivation

The Index of Multiple Deprivation (IMD) decile measures from 2019 [42] were extracted for information on neighbourhood deprivation for each patient, based on their address at the time of the index date aggregated by Lower Super Output Area (LSOA) - a standard national administrative unit containing an average 1500 residents. National Census data are used to calculate IMD scores for each LSOA. A lower IMD decile indicates higher deprivation levels. Individuals with missing IMD scores were retained in a separate category.

Recorded Pain

Pain-related keywords generated from a lexicon of pain terms [43] were used to identify patients in the cohort who had mentions of physical pain (such as “worsening back pain”, “suffers from headaches”, “complains of pain”) recorded in their clinical notes within the predetermined window. The lexicon contains terms such as aching muscles, backpain, headache, myalgia, etc and can be accessed online[1]. An NLP application (F1-score, i.e., harmonic mean of precision and recall: 0.98) was used on the documents of these patients. The application was developed by fine-tuning an existing model (SapBERT [44]) with 5,644 gold standard annotations (triple annotated by medical student annotators) from CRIS, with the intention of classifying sentences within documents as relevant or not, where relevant refers to a mention of physical pain affecting the patient, such as “complained of pain”, “has muscle aches..”, and not relevant refers to no or negated mentions, hypothetical mentions, and metaphorical mentions of pain, such as “..defensive of painful feelings”, and “..painful consequences of using alcohol”. Only relevant mentions were used in the results reported here. The application has been described in detail in [45].

As with all other UK research based on access to anonymised primary care records, LDN does not allow access to any free text clinical notes. For this reason, pain information can only be extracted from the structured fields of the records. Read codes [46] were used to identify patients who had a pain diagnosis or were on any pain medications and treatments:

---

[1]https://docs.google.com/spreadsheets/d/1z-6619UBdvwWrB9Sz4b1rbjDzuslOGCpts2DNc0naCc/edit?usp=sharing

For peer review only

1. Pain medications code list - developed as part of a project described in [47], which focused on analgesics (obtained from dm+d (a dictionary of medicines and devices [48]) used in the treatment of 35 long-term conditions. These 35 conditions were obtained from [49], a cross-sectional study on multimorbidities in patients registered with 314 medical practices in Scotland as of March 2007.
2. Pain diagnosis and treatments code list - developed as part of a collaboration project with Outcomes Based Healthcare (OBH), an organisation that provides a platform for the study of population health outcomes [50], with the research described in [51]. Pain diagnosis codes included instances such as back pain, referred ear pain, arthritis, and trigeminal neuralgia. Pain treatment codes included codes for referral to pain clinic, seen in pain clinic, and under the care of pain management specialist.

While these codes were developed for chronic pain, they are generic enough to be used for this research, as highlighted in the examples mentioned. These code lists are available on GitHub[2].

Anatomy Related to Recorded Pain

Another NLP application was developed as part of this research for identifying anatomy mentioned in relation to pain. This was a sentence classifier that generated a binary output - “mentioned” or “not mentioned”. The application was trained on 4,026 gold standard sentences about anatomy mentioned in relation to pain, and performed with an F1 score of 0.94. These gold standard sentences were a subset of the sentences used to train the pain NLP application. This application was run on sentences labelled as relevant by the pain application. Once the sentences that contained mentions of body parts were identified, they were run through MedCATTrainer [52], which used named entity recognition (NER), a type of NLP task to label entities within the text to identify the specific body parts mentioned within the text. The purpose of using MedCATTrainer was that it linked the identified body parts to unique identification numbers (SCTID) from SNOMED CT, a terminology of clinical terms. These SCTIDs were used to aggregate the mentioned body parts, for ease of analysis. For example, foot, calf, and knee mentions would be aggregated under “lower limb”.

Overlap between CRIS and LDN

To examine the overlap across primary (LDN) and secondary (CRIS) care, the patient IDs from the CRIS cohort (N=27,211) were searched for matching records within the LDN database over the same window of July 1, 2017 to July 1, 2019. Variables were generated indicating the presence of the patients within LDN, along with variables indicating the presence of any codes for pain medication, diagnosis or treatment based on the predefined lists described above. This allowed the identification of patients with documented pain experiences in both their mental health and primary care records for the aligned time period. The cross-referencing process enabled the comparison of recorded pain between the two systems at the patient level.

[2] https://github.com/jayachaturvedi/pain_in_mental_health

For peer review only

Descriptive Statistics

All analysis was conducted using STATA v15.1 and the Python programming language (version 3.10.0).

Descriptive statistics were obtained for demographic, deprivation and diagnosis features and compared between the two groups - patients who had recorded pain (class 1) and those who did not (class 0) - within their clinical notes. Chi-squared tests and logistic regression were conducted between the two classes to obtain adjusted odds ratios. Frequencies of body parts affected by pain and the overlap of recorded pain experiences between CRIS and LDN were also reported.

Results

Data Extraction

Based on the extraction criteria, 27,211 patients were represented. Amongst these patients, 18,188 had pain keywords mentioned within their documents. These documents were run through the NLP application to label them as relevant to pain (class 1) or not (class 0), resulting in 14,202 patients who had relevant mentions of pain within their clinical notes (Figure 1). Relevant mentions include instances such as “complains of pain”, “experiencing headaches”, “worsening back pain”, and “has stomach cramps”. Mentions that were not relevant were instances such as negations (“denied pain..”, “no complaint of pain”), mentions within forms (“..experiencing other physical symptoms? E.g. chest pain”, misspelt words (“..pained and decorated the walls”), hypothetical mentions (“ reduce risk of pressure sores”, “fear that eating will cause throat pain”), and metaphorical mentions (“life is too painful to carry on living”, “pain will end when she repents”).

<Figure 1>

Cohort Characteristics and Pain Mentions

Amongst the cohort of 27,211 patients, the mean age of the cohort was 44 (Inter-quartile range 29-55, SD 17.5), with 50.3% female and 48.2% of White ethnicity. The majority of the cohort (72.2%) lived in more deprived areas (IMD score <=5), and 67.0% received a non-SMI diagnosis. 66.8% of the patients (18,188 patients and 174,167 mentions within documents) contained pain keywords within their documents, and 52.1% of the cohort (14,202 patients) contained relevant mentions of pain in their documents.

Records of 52.1% of the patients within the cohort contained relevant mentions of pain. Differences between the patients who showed recorded pain (class 1) in their clinical notes and those who didn’t (class 0) are shown in Table 1. Class 0 includes patients who did not have any pain mentions in their documents, as well as patients whose pain mentions were classified as not relevant. Patients within class 1 had a mean of10 pain mentions per document (median = 4).

For peer review only

Characteristic	n	Class 0 (no recorded pain)	Class 1 (recorded pain)
N (%)	27,211	13,009 (47.9)	14,202 (52.1)
Mean Age (IQR)	44 (29–55)	41 (27–52)	46 (32–56)
Gender (N, %)			
Male	13,471	7,037 (54.1)	6,434 (45.3)
Female	13,709	5,953 (45.7)	7,756 (54.6)
Not known	31	19 (0.2)	12 (0.1)
Ethnicity (N, %)			
White	13,139	6,014 (46.2)	7,125 (50.1)
Black	5,866	2,115 (16.2)	3,751 (26.4)
Not stated/known	4,708	3,418 (26.2)	1,290 (9.0)
Asian	1,506	592 (4.5)	914 (6.4)
Other	1,197	512 (3.9)	685 (4.8)

For peer review only - http://bmjopen.bmj.com/site/about/guidelines.xhtml

Mixed	795	358 (2.7)	437 (3.0)
Index of Multiple Deprivation (N, %) Decile 2019			
<= 5 (more deprived)	19,660	8,847 (68.0)	10,813 (76.1)
> 5 (less deprived)	6,686	3,836 (29.4)	2,850 (20.0)
Not known	865	326 (2.5)	539 (3.9)
Primary Diagnosis: SMI vs Non-SMI (ICD-9 code) (N, %)			
SMI	8,962	3,059 (23.5)	5,903 (41.5)
Non-SMI	18,249	9,950 (76.5)	8,299 (58.5)

Table 1. Distributions between the two classes - class 0 (no recorded pain) and class 1 (recorded pain)

Demographic variations emerged between those with/without recorded pain in the cohort, as shown in Table 1. The mean age was higher in patients with recorded pain at 46 (SD=17) compared to 41 (SD=17) for the remainder. Patients with recorded pain were more likely to be female and had a higher representation across all ethnic minorities. Additionally, patients with documented pain experiences were more likely to live in higher deprivation neighbourhoods.

Table 2 presents demographic, deprivation and diagnostic associations with recorded pain obtained through logistic regressions (unadjusted and adjusted for different factors as detailed below).

	Logistic Regression models				
	Unadjusted	Mutually adjusted			
		Model 1	Model 2	Model 3	Model 4
**Age** (per 10 years)	1.17 [1.15, 1.19] *	1.12 [1.11, 1.14] *	1.12 [1.11, 1.14] *	1.11 [1.10, 1.13] *	-

For peer review only

**Gender**					
Male	1 (reference)	1 (reference)	1 (reference)	1 (reference)	-
Female	1.42 [1.35, 1.49] *	1.42 [1.35, 1.49] *	1.43 [1.36, 1.50] *	1.47 [1.40, 1.55] *	-
Not known	0.69 [0.33, 1.42]	1.08 [0.50, 2.33]	1.06 [0.49, 2.30]	1.10 [0.51, 2.38]	-
**Ethnicity**					
White	1 (reference)	1 (reference)	1 (reference)	1 (reference)	1 (reference)
Asian	1.30 [1.16, 1.45] *	1.36 [1.22, 1.52] *	1.34 [1.19, 1.49] *	1.21 [1.08, 1.36] *	1.29 [1.15, 1.44] *
Black	1.49 [1.40, 1.59] *	1.58 [1.48, 1.69] *	1.50 [1.40, 1.60] *	1.25 [1.17, 1.34]	1.42 [1.33, 1.52] *
Other	1.12 [1.00, 1.27]	1.20 [1.06, 1.36]	1.17 [1.03, 1.32]	1.10 [0.97, 1.24]	1.08 [0.96, 1.33]
Mixed	1.03 [0.89, 1.18]	1.15 [0.99, 1.33]	1.12 [0.96, 1.30]	1.06 [0.91, 1.23]	1.01 [0.87, 1.17]
Not known	0.31 [0.29, 0.34] *	0.36 [0.34, 0.39] *	0.37 [0.34, 0.40] *	0.40 [0.37, 0.44] *	0.32 [0.30, 0.35] *
**Index of Multiple Deprivation**					
National Decile <=5	1.64 [1.55, 1.73] *	-	1.43 [1.35, 1.51] *	1.37 [1.29, 1.45] *	1.41 [1.33, 1.50] *
**Diagnosis**					
SMI	0.43 [0.41, 0.46] *	-	-	0.56 [0.53, 0.59] *	-

Table 2. Logistic Regression findings for variables reflecting differences in class 0 (no recorded pain) and class 1 (recorded pain) (N = 27,211). Outcome is recorded pain vs no recorded pain.
Values are given as odds ratio (95% CI), and * indicates significance at $p < 0.001$
Model 1 contained the demographic variables only [age, gender and ethnicity].
Model 2 contained the variables from Model 1, plus the variable for deprivation (IMD Decile].
Model 3 contained the variables from Model 2 plus the diagnosis variable.
Model 4 contains the ethnicity and deprivation variables alone.

Unadjusted odds ratios revealed patients with documented pain experiences were more likely to be older (OR 1.17, 95% CI 1.15-1.19, p<0.001), female (OR 1.42, 95% CI 1.35-1.49, p<0.001), of Asian (OR 1.30 in relation to a White reference group, 95% CI 1.16-1.45, p<0.001) or Black (OR 1.49, 95% CI 1.40-1.59, p<0.001) ethnicities, and living in deprived neighbourhoods (OR 1.64, 95% CI 1.55-1.73, p<0.001) when compared to the respective reference groups. In a model containing all demographic variables (Model 1), the odds ratios for documented pain remained significant for all ethnic minority groups compared to the White group. With additional adjustment for neighbourhood deprivation (Model 2), the odds ratios

For peer review only

were still significant for females relative to males. Similarly, in the model also adjusted for diagnoses (Model 3), the odds ratios were also significant for females versus males. Patients with SMI had lower odds of documented pain (OR 0.43, 95% CI 0.41-0.46, p<0.001) than non-SMI patients, which stayed significant after accounting for demographics, deprivation and diagnosis (Model 3). A supplementary model (Model 4) including both ethnicity and deprivation as covariates showed increased odds for Asian and Black ethnicities when compared to White patients, and those in more deprived neighbourhoods. The motivation for this model was to disentangle the independent contributions of ethnicity and deprivation to the differences in pain documentation. By adjusting for deprivation while evaluating the association between ethnicity and recorded pain (and vice versa), we can derive better effect estimates for each factor. This approach helps us to understand whether ethnicity-related differences persist after accounting for socioeconomic factors. We present selected incremental models for transparency in how estimates shifted with inclusion of covariates, but focus our interpretation on the unadjusted and fully adjusted model 3, which highlight the patterns with most clarity.

Anatomy Distributions

Additional descriptive data were generated on the anatomical location of the pain reported. Amongst the 14,202 patients with any recorded pain, there were 174,167 mentions of pain within the documents. Of these, 7,555 (53%) patients included 40,418 mentions of the anatomy associated with the pain. Of these 53%, each patient had an average of 5 body parts mentioned in the context of pain. The most common body part affected by pain, as per the recorded mentions, was lower limbs, which accounted for 20% of all mentions where anatomy could be ascertained (Table 3).

**Body Part**	**Mentions**	**Frequency (mention-level)**
Lower limbs	Feet, ankle, leg, knee, calf, thigh, toes	20%
Upper body, excluding back	Chest, side of chest, upper body, torso	19%
Upper limbs	Hand, wrist, arm, elbow, thumb, shoulder	17%
Stomach/abdomen region	Stomach, abdomen, groin, bladder, prostate	16%
Head and neck	Head, tooth, face, mouth, tongue, eye, ear, neck	15%
Non-specific site	Entire body, skin, muscle, joint	8%

Back	Back, lower back	5%

Table 3. Body parts affected (at mention level)

Similar distributions were found within the SMI and non-SMI groups. Amongst patients with an SMI diagnosis, the most frequent body parts mentioned were lower limbs (23%), upper body, excluding back (17%) and stomach/abdomen region (15%). Patients with a non-SMI diagnosis most frequently reported lower limbs (19%), stomach/abdomen region (18%) and upper body, excluding back (17%), with minor variations in the frequencies.

Overlap with Primary Care

When comparing secondary care CRIS records with those of primary care from LDN, among the 27,211 patients of the CRIS cohort, 4,822 patients (17%) also had records in LDN. Amongst these patients who had records in both CRIS and LDN, 1,507 (31%) patients were identified as having some recorded instance of pain in both their records, while 687 (14%) patients showed recorded pain only in LDN (primary care). Among the 27,211 patients within CRIS, 12,695 (46%) had recorded pain only within CRIS (mental health care), as seen in Figure 2.

<Figure 2>

Discussion

This study investigated the differences observed in recorded pain mentions within the clinical notes of mental health records. The results reflect current literature findings that pain is a common issue among patients with mental health disorders. In a cohort of 27,211 patients, 18,188 (67%) patients contained pain-related keywords in their text, and 14,202 (52%) patients had relevant pain mentions, i.e., the mention indicated physical pain affecting the patient in question. We found differences in documented pain mentions across genders, with a greater proportion recorded among female patients. This aligns with previous literature demonstrating gender differences in pain reporting and experiences [16,48,49,53,54]. Furthermore, while patients with known ethnicities had higher frequencies of recorded pain in the cohort of relevant pain mentions (in relation to those with unknown ethnicity), most noticeable were the Black, Asian and other ethnic groups. This highlights the need for a comprehensive exploration of pain experiences across diverse populations [55]. Moreover, the study's findings are also consistent with studies that indicate the impact of deprivation on health outcomes [21], as people living in more deprived areas (IMD decile <= 5) were more frequently recorded with pain.

When comparing the overlap of patients between primary and secondary care, it was found that 17% of the patients within the CRIS cohort also had records within LDN. Amongst these patients, 31% had recorded pain instances in both records. While this overlap between primary and secondary care seems low, it is important to bear in mind that Lambeth only represents

22% of the catchment covered by CRIS [56]. Patients present in CRIS but not in LDN could include patients who have recorded instances of pain within the free-text clinical notes in LDN and might have been missed in this study since we do not have access to this text. Furthermore, this study did not differentiate between acute and chronic pain mentions and focused on extracting mentions of physical pain of any duration. As a result, the higher occurrence of pain mentioned within CRIS can be partially attributed to the documentation of such acute or short-lived pain episodes. Conversely, the GP records within LDN likely focus on recording persistent and chronic pain experiences. This disparity in recording pain should be considered when interpreting the findings of this study. Looking specifically at chronic pain instances within the CRIS notes may improve the comparability. However, the temporal information required to determine pain chronicity from clinical notes is a particular challenge and can be difficult to extract reliably. Future work can attempt to differentiate acute and chronic pain through temporal or contextual information, which could provide richer insight. However, the current broad inclusion of pain provides wider coverage for this initial exploration of pain mentioned within clinical notes.

The findings of this study highlight important considerations that need to be made regarding the assessment and management of pain among people with SMI. Existing literature demonstrates that individuals with SMI often underreport pain symptoms yet experience disproportionately high rates of chronic pain conditions compared to the general population [57,58]. The low documentation of pain in the mental health records of this cohort indicates potential gaps in detection that warrant attention, particularly given research showing links between untreated pain and worse mental health outcomes. The challenges faced by this group in communicating their pain may inhibit pain identification [59]. Additional training focused on regular, thorough pain assessment within this group is needed for mental health professionals. Specifically, potentially implementing structured screening protocols, allowing patient self-report through diverse modalities, and increased awareness of typical presentations could improve documentation and care standards. Pain assessment should become a routine aspect of comprehensive care for those with SMI to reduce compounding health decline. These steps toward more patient-centred pain management align with calls to better integrate physical and mental health services for this vulnerable population.

A strength of this study is the size of the data set available and the access to information about pain from the clinical text. To the best of our knowledge, this is potentially the first cross-sectional study to summarise and describe the distribution of recorded pain derived from routine mental health records. While the cohort data extraction did not apply any filters on demographics, aiming for broad representativeness, other systemic biases related to access to healthcare resources may still exist. Factors like deprivation level and ethnicity can influence the utilisation of services and, therefore, documentation within health records, often stemming from perceived barriers to access. However, by not restricting cohort selection on demographic factors, this study intended to capture a diverse patient population receiving care across the South London boroughs.

A limitation of this study is its reliance on clinician documentation of pain within the clinical notes, which may be subject to a form of reporting bias. Specifically, the absence of documented pain could either be because patients were asked about their pain and this information wasn't recorded, or because the patients were never asked about their pain. Absence of recorded pain does not indicate that the patients were not experiencing pain or

For peer review only

that clinicians did not inquire about pain. This study methodology does not distinguish between these scenarios. The actual occurrences of pain experiences could remain unaccounted for if they weren't recorded by the clinicians or were not shared with the clinicians, especially for patients with severe mental illnesses who might be completely or partially nonverbal. While the NLP application achieved good performance metrics during its development and evaluation [39], it is not impervious to imperfections. Instances of pain experiences might have been overlooked if they were not included as examples during the training of the application.

The scope of this study is limited to the examination of mental health records from an EHR database in South London. It is essential to acknowledge the potential influence of gender and ethnicity on the reporting of pain experiences, particularly if females and minority ethnicities (due to language barriers or other reasons) are less likely to self-report their pain experiences [55,60,61]. Since the focus of this study has been on a mental health EHR database, the clinical care within this setting is focused on mental health issues reported by the patients. Consequently, as much importance might not be given to the investigation and reporting of physical health conditions such as pain.

Conclusion

The outcomes of this study have significant implications for the assessment and management of pain amongst patients with mental health disorders and highlight the importance of utilising NLP methods on EHR databases for research purposes. Notably, these findings reiterate the recommendations set forth by Mental Health America [62], advocating the need for proactive initiation of conversations around mental health and pain with patients. Relying solely on patients to self-report symptoms could potentially lead to worse outcomes, especially since the stigma surrounding persistent pain and mental health conditions may prevent patients from seeking the necessary treatment. Thus, early and proactive interventions could go a long way towards improved long-term outcomes. Unfortunately, there still exists a perceived lack of credibility and empathy towards patients living with pain [63], particularly when compounded by co-existent mental illnesses. This was one of the main points shared by the PPI group consulted as part of this study. More research in this area can help towards these issues and provide safer and equitable access to good-quality pain management.

It is possible that some patients within the cohort, and in general within the CRIS database, might be receiving psychological therapies for persistent pain. Future research leveraging the LDN-CRIS data linkage could examine referral patterns to these services. Examining referral trends over time and across demographic factors may uncover important insights regarding access barriers, and ultimately enhance delivery of appropriate psychological care for those suffering from pain. Analysing such patterns of psychological therapy referrals using the LDN-CRIS linked data can expand our understanding of this dimension of care.

While these findings represent a step forward, they are only one side of the story. Combining these findings with patient-reported insights could offer a more comprehensive understanding of pain experiences within this cohort. However, achieving this is a challenging task due to the lack of such data and the inability to link patient-reported experiences to their health records. Further research is needed to better understand the relationship between pain and mental

For peer review only

health in this population. This could be achieved by accessing longitudinal data within this database, and studying the temporal aspects of both conditions.

Author Contributions

The idea was conceived by JC, AR, and RS. JC conducted the analyses and drafted the manuscript. MA provided insights on LDN data. AR and RS provided guidance in the design and interpretation of results. All authors commented on drafts of the manuscript and approved the final version.

Competing Interests

This paper represents independent research part-funded by the National Institute for Health Research (NIHR) Biomedical Research Centre at South London and Maudsley NHS Foundation Trust and King's College London. The views expressed are those of the authors and not necessarily those of the NHS, the NIHR or the Department of Health and Social Care. The funders had no role in study design, data collection and analysis, decision to publish, or preparation of the manuscript.

Funding

AR is funded by Health Data Research UK, an initiative funded by UK Research and Innovation, Department of Health and Social Care (England) and the devolved administrations, and leading medical research charities. RS is part-funded by i) the National Institute for Health Research (NIHR) Biomedical Research Centre at South London and Maudsley NHS Foundation Trust and King's College London; ii) the National Institute for Health Research (NIHR) Applied Research Collaboration South London (NIHR ARC South London) at King's College Hospital NHS Foundation Trust; iii) the DATAMIND HDR UK Mental Health Data Hub (MRC grant MR/W014386); iv) the UK Prevention Research Partnership (Violence, Health and Society; MR-VO49879/1), an initiative funded by UK Research and Innovation Councils, the Department of Health and Social Care (England) and the UK devolved administrations, and leading health research charities. JC is supported by the KCL-funded Centre for Doctoral Training (CDT) in Data-Driven Health. The funders were not involved in the study design, collection, analysis, interpretation of data, the writing of this article or the decision to submit it for publication. RS declared research support received in the last 36 months from Janssen, GSK and Takeda. All other authors declare no other competing interests.

This work uses data provided by patients and collected by the NHS as part of their care and support. An application for access to the Clinical Record Interactive Search (CRIS) database for this project was submitted and approved by the CRIS Oversight Committee. The authors would like to acknowledge Dr Ruimin Ma for her help in obtaining the LDN codes.

For peer review only

Data Sharing Statement

Data are owned by a third party, Maudsley Biomedical Research Centre (BRC) Clinical Records Interactive Search (CRIS) tool, which provides access to anonymised data derived from SLaM electronic medical records. These data can be accessed by permitted individuals from within a secure firewall (i.e. the data cannot be sent elsewhere) in the same manner as the authors. For more information, please contact cris.administrator@slam.nhs.uk. Any STATA and Python code used in this project will be available on GitHub[3].

Ethics Approval Statement

CRIS (as well as its associated linkages) has received ethical approval as a data resource for secondary analysis from the Oxford C Research Ethics Committee (reference 23/SC/0257). A patient-led oversight committee (detailed in [39]) reviews and approves research projects that use the CRIS database. For service users, an opt-out system is in place and is advertised in all promotional materials and campaigns. Only authorised individuals can access this data from within a secure firewall. The CRIS project approval references for this work are 21-021 and 23-003.

LDN approval was obtained as part of an existing CRIS project (project number 23-124) which included access to linked data from LDN (Caldicott Guardian approval, 15/9/21). This CRIS-LDN project aimed to examine the profile of patients with mental illnesses and chronic/persistent pain and compare them to controls from LDN who had chronic/persistent pain only.

Bibliography

1 Phillips CJ. The cost and burden of chronic pain. *Rev Pain* 2009;**3**:2–5. doi:10.1177/204946370900300102

2 Enright A, Goucke R. The global burden of pain: the tip of the iceberg? *Anesth Analg* 2016;**123**:529–30. doi:10.1213/ANE.0000000000001519

3 Merlin JS, Zinski A, Norton WE, *et al.* A conceptual framework for understanding chronic pain in patients with HIV. *Pain Pract* 2014;**14**:207–16. doi:10.1111/papr.12052

4 Meints SM, Edwards RR. Evaluating psychosocial contributions to chronic pain outcomes. *Prog Neuropsychopharmacol Biol Psychiatry* 2018;**87**:168–82. doi:10.1016/j.pnpbp.2018.01.017

5 Bridges S. Chapter 2: Mental health problem. In: *Health Survey*. 2014.

6 Abplanalp SJ, Mueser KT, Fulford D. The role of physical pain in global functioning of people with serious mental illness. *Schizophr Res* 2020;**222**:423–8. doi:10.1016/j.schres.2020.03.062

7 Public Health England. Severe mental illness (SMI) and physical health inequalities:

[3] https://github.com/jayachaturvedi/pain_in_mental_health

For peer review only

briefing. Research and analysis. 2018.https://www.gov.uk/government/publications/severe-mental-illness-smi-physical-health-inequalities/severe-mental-illness-and-physical-health-inequalities-briefing#:~:text=The%20phrase%20severe%20mental%20illness,an%20SMI%20%5Bfootnote%201%5D. (accessed 23 Jun2023).

8 Stubbs B, Mitchell AJ, De Hert M, *et al.* The prevalence and moderators of clinical pain in people with schizophrenia: a systematic review and large scale meta-analysis. *Schizophr Res* 2014;**160**:1–8. doi:10.1016/j.schres.2014.10.017

9 Bonnot O, Anderson GM, Cohen D, *et al.* Are patients with schizophrenia insensitive to pain? A reconsideration of the question. *Clin J Pain* 2009;**25**:244–52. doi:10.1097/AJP.0b013e318192be97

10 Birgenheir DG, Ilgen MA, Bohnert ASB, *et al.* Pain conditions among veterans with schizophrenia or bipolar disorder. *Gen Hosp Psychiatry* 2013;**35**:480–4. doi:10.1016/j.genhosppsych.2013.03.019

11 Potvin S, Marchand S. Hypoalgesia in schizophrenia is independent of antipsychotic drugs: a systematic quantitative review of experimental studies. *Pain* 2008;**138**:70–8. doi:10.1016/j.pain.2007.11.007

12 IsHak WW, Wen RY, Naghdechi L, *et al.* Pain and depression: A systematic review. *Harv Rev Psychiatry* 2018;**26**:352–63. doi:10.1097/HRP.0000000000000198

13 Bair MJ, Robinson RL, Katon W, *et al.* Depression and pain comorbidity: a literature review. *Arch Intern Med* 2003;**163**:2433–45. doi:10.1001/archinte.163.20.2433

14 Blumer D, Heilbronn M. Chronic pain as a variant of depressive disease: the pain-prone disorder. *J Nerv Ment Dis* 1982;**170**:381–406. doi:10.1097/00005053-198207000-00001

15 Rayner L, Hotopf M, Petkova H, *et al.* Depression in patients with chronic pain attending a specialised pain treatment centre: prevalence and impact on health care costs. *Pain* 2016;**157**:1472–9. doi:10.1097/j.pain.0000000000000542

16 Thompson T, Correll CU, Gallop K, *et al.* Is Pain Perception Altered in People With Depression? A Systematic Review and Meta-Analysis of Experimental Pain Research. *J Pain* 2016;**17**:1257–72. doi:10.1016/j.jpain.2016.08.007

17 Noroozian M, Raeesi S, Hashemi R, *et al.* Pain: the neglect issue in old people's life. *Open Access Maced J Med Sci* 2018;**6**:1773–8. doi:10.3889/oamjms.2018.335

18 Fillingim RB. Sex, gender, and pain. In: *Principles of Gender-Specific Medicine*. Elsevier 2017. 481–96. doi:10.1016/B978-0-12-803506-1.00038-3

19 Vallerand AH, Polomano RC. The relationship of gender to pain. *Pain Manag Nurs* 2000;**1**:8–15. doi:10.1053/jpmn.2000.9759

20 Campbell CM, Edwards RR. Ethnic differences in pain and pain management. *Pain Manag* 2012;**2**:219–30. doi:10.2217/pmt.12.7

21 Martin JL, McLean G, Park J, *et al.* Impact of socioeconomic deprivation on rate and cause of death in severe mental illness. *BMC Psychiatry* 2014;**14**:261. doi:10.1186/s12888-014-0261-4

22 Lambert TJR, Newcomer JW. Are the cardiometabolic complications of schizophrenia still neglected? Barriers to care. *Med J Aust* 2009;**190**:S39-42. doi:10.5694/j.1326-

For peer review only

5377.2009.tb02374.x

23 DE Hert M, Correll CU, Bobes J, *et al.* Physical illness in patients with severe mental disorders. I. Prevalence, impact of medications and disparities in health care. *World Psychiatry* 2011;**10**:52–77. doi:10.1002/j.2051-5545.2011.tb00014.x

24 Frayne SM, Halanych JH, Miller DR, *et al.* Disparities in diabetes care: impact of mental illness. *Arch Intern Med* 2005;**165**:2631–8. doi:10.1001/archinte.165.22.2631

25 Laursen TM, Munk-Olsen T, Agerbo E, *et al.* Somatic hospital contacts, invasive cardiac procedures, and mortality from heart disease in patients with severe mental disorder. *Arch Gen Psychiatry* 2009;**66**:713–20. doi:10.1001/archgenpsychiatry.2009.61

26 Aro S, Aro H, Keskimäki I. Socio-economic mobility among patients with schizophrenia or major affective disorder. A 17-year retrospective follow-up. *Br J Psychiatry* 1995;**166**:759–67. doi:10.1192/bjp.166.6.759

27 Barnett P, Oshinowo I, Cooper C, *et al.* The association between social class and the impact of treatment for mental health problems: a systematic review and narrative synthesis. *Soc Psychiatry Psychiatr Epidemiol* 2023;**58**:581–603. doi:10.1007/s00127-022-02378-9

28 Lawrence D, Kisely S. Inequalities in healthcare provision for people with severe mental illness. *J Psychopharmacol (Oxford)* 2010;**24**:61–8. doi:10.1177/1359786810382058

29 Velupillai S, Suominen H, Liakata M, *et al.* Using clinical Natural Language Processing for health outcomes research: Overview and actionable suggestions for future advances. *J Biomed Inform* 2018;**88**:11–9. doi:10.1016/j.jbi.2018.10.005

30 Kadra G, Stewart R, Shetty H, *et al.* Long-term antipsychotic polypharmacy prescribing in secondary mental health care and the risk of mortality. *Acta Psychiatr Scand* 2018;**138**:123–32. doi:10.1111/acps.12906

31 Bendayan R, Kraljevic Z, Shaari S, *et al.* Mapping multimorbidity in individuals with schizophrenia and bipolar disorders: evidence from the South London and Maudsley NHS Foundation Trust Biomedical Research Centre (SLAM BRC) case register. *BMJ Open* 2022;**12**:e054414. doi:10.1136/bmjopen-2021-054414

32 Jackson RG, Patel R, Jayatilleke N, *et al.* Natural language processing to extract symptoms of severe mental illness from clinical text: the Clinical Record Interactive Search Comprehensive Data Extraction (CRIS-CODE) project. *BMJ Open* 2017;**7**:e012012. doi:10.1136/bmjopen-2016-012012

33 Sampson R, Cooper J, Barbour R, *et al.* Patients' perspectives on the medical primary-secondary care interface: systematic review and synthesis of qualitative research. *BMJ Open* 2015;**5**:e008708. doi:10.1136/bmjopen-2015-008708

34 Benchimol EI, Smeeth L, Guttmann A, *et al.* [The REporting of studies Conducted using Observational Routinely-collected health Data (RECORD) statement]. *Z Evid Fortbild Qual Gesundhwes* 2016;**115–116**:33–48. doi:10.1016/j.zefq.2016.07.010

35 von Elm E, Altman DG, Egger M, *et al.* Strengthening the Reporting of Observational Studies in Epidemiology (STROBE) statement: guidelines for reporting observational studies. *BMJ* 2007;**335**:806–8. doi:10.1136/bmj.39335.541782.AD

36 Stewart R, Soremekun M, Perera G, *et al.* The South London and Maudsley NHS

For peer review only

Foundation Trust Biomedical Research Centre (SLAM BRC) case register: development and descriptive data. *BMC Psychiatry* 2009;**9**:51. doi:10.1186/1471-244X-9-51

37 N H S. Lambeth DataNet. Lambeth DataNet. 2021.https://selondonccg.nhs.uk/wp-content/uploads/2021/04/Lambeth-DataNet.pdf (accessed 26 May2023).

38 NIHR Maudsley Biomedical Research Center. CRIS Data Linkages. CRIS Data Linkages. https://www.maudsleybrc.nihr.ac.uk/facilities/clinical-record-interactive-search-cris/cris-data-linkages/ (accessed 7 Aug2023).

39 Fernandes AC, Cloete D, Broadbent MTM, *et al.* Development and evaluation of a de-identification procedure for a case register sourced from mental health electronic records. *BMC Med Inform Decis Mak* 2013;**13**:71. doi:10.1186/1472-6947-13-71

40 Research Design Service South Central. Patient and Public Involvement (PPI). What is Patient and Public Involvement in health and social care research? 2023.https://www.rds-sc.nihr.ac.uk/ppi-information-resources/

41 World Health Organization. International Classification of Diseases (ICD). 2008.https://www.who.int/standards/classifications/classification-of-diseases (accessed 7 Aug2023).

42 GOV.UK. English indices of deprivation 2019. 2019.https://www.gov.uk/government/statistics/english-indices-of-deprivation-2019 (accessed 7 Aug2023).

43 Chaturvedi J, Mascio A, Velupillai SU, *et al.* Development of a lexicon for pain. *Front Digit Health* 2021;**3**:778305. doi:10.3389/fdgth.2021.778305

44 Liu F, Shareghi E, Meng Z, *et al.* Self-Alignment Pretraining for Biomedical Entity Representations. *arXiv:201011784 [cs]* 2021.

45 Chaturvedi J, Velupillai S, Stewart R, et al. Identifying mentions of pain in mental health records text: A natural language processing approach. Stud Health Technol Inform 2024;310:695–9. doi:10.3233/SHTI231054

46 NHS Digital. Read Codes. 2022.https://digital.nhs.uk/services/terminology-and-classifications/read-codes (accessed 7 Aug2023).

47 Ma R, Romano E, Ashworth M, *et al.* Multimorbidity clusters among people with serious mental illness: a representative primary and secondary data linkage cohort study. *Psychol Med* 2022;:1–12. doi:10.1017/S003329172200109X

48 NHSBSA. Dictionary of medicines and devices (dm+d) . 2023.https://www.nhsbsa.nhs.uk/pharmacies-gp-practices-and-appliance-contractors/dictionary-medicines-and-devices-dmd (accessed 2 Aug2023).

49 Barnett K, Mercer SW, Norbury M, *et al.* Epidemiology of multimorbidity and implications for health care, research, and medical education: a cross-sectional study. *Lancet* 2012;**380**:37–43. doi:10.1016/S0140-6736(12)60240-2

50 Outcomes Based Healthcare. Outcomes Platform. 2018.https://outcomesbasedhealthcare.com/outcomes-platform/ (accessed 3 Aug2023).

51 Hafezparast N, Bragan Turner E, Dunbar-Rees R, *et al.* Identifying populations with chronic pain in primary care: developing an algorithm and logic rules applied to coded primary care diagnostic and medication data. *BMC Primary Care* 2023.

For peer review only

52 Searle T, Kraljevic Z, Bendayan R, *et al.* MedCATTrainer: A Biomedical Free Text Annotation Interface with Active Learning and Research Use Case Specific Customisation. In: *Proceedings of the 2019 Conference on Empirical Methods in Natural Language Processing and the 9th International Joint Conference on Natural Language Processing (EMNLP-IJCNLP): System Demonstrations*. Stroudsburg, PA, USA: : Association for Computational Linguistics 2019. 139–44. doi:10.18653/v1/D19-3024

53 Wandner LD, Scipio CD, Hirsh AT, *et al.* The perception of pain in others: how gender, race, and age influence pain expectations. *J Pain* 2012;**13**:220–7. doi:10.1016/j.jpain.2011.10.014

54 Fillingim RB. Sex, gender, and pain: women and men really are different. *Curr Rev Pain* 2000;**4**:24–30. doi:10.1007/s11916-000-0006-6

55 Green CR, Anderson KO, Baker TA, *et al.* The unequal burden of pain: confronting racial and ethnic disparities in pain. *Pain Med* 2003;**4**:277–94. doi:10.1046/j.1526-4637.2003.03034.x

56 Perera G, Broadbent M, Callard F, *et al.* Cohort profile of the South London and Maudsley NHS Foundation Trust Biomedical Research Centre (SLaM BRC) Case Register: current status and recent enhancement of an Electronic Mental Health Record-derived data resource. *BMJ Open* 2016;**6**:e008721. doi:10.1136/bmjopen-2015-008721

57 Brooks JM, Polenick CA, Bryson W, *et al.* Pain intensity, depressive symptoms, and functional limitations among older adults with serious mental illness. *Aging Ment Health* 2019;**23**:470–4. doi:10.1080/13607863.2017.1423025

58 Engels G, Francke AL, van Meijel B, *et al.* Clinical pain in schizophrenia: a systematic review. *J Pain* 2014;**15**:457–67. doi:10.1016/j.jpain.2013.11.005

59 Onwumere J, Stubbs B, Stirling M, *et al.* Pain management in people with severe mental illness: an agenda for progress. *Pain* 2022;**163**:1653–60. doi:10.1097/j.pain.0000000000002633

60 Samulowitz A, Gremyr I, Eriksson E, *et al.* “Brave Men” and “Emotional Women”: A Theory-Guided Literature Review on Gender Bias in Health Care and Gendered Norms towards Patients with Chronic Pain. *Pain Res Manag* 2018;**2018**:6358624. doi:10.1155/2018/6358624

61 Hoffmann DE, Tarzian AJ. The girl who cried pain: a bias against women in the treatment of pain. *J Law Med Ethics* 2001;**29**:13–27. doi:10.1111/j.1748-720X.2001.tb00037.x

62 American Psychiatric Association. Chronic Pain and Mental Health Often Interconnected. 2020.https://www.psychiatry.org/news-room/apa-blogs/chronic-pain-and-mental-health-interconnected (accessed 3 Aug2023).

63 Bennett C. What you need to know about pain and mental health in Australia. Pain Australia. 2020.https://www.painaustralia.org.au/media-document/blog-1/blog-2020/blog-2019/what-you-need-to-know-about-pain-and-mental-health-in-australia (accessed 3 Aug2023).

For peer review only

Figure Legend

Figure 1 – Data Extraction
Figure 2 – Overlap of recorded pain in CRIS and LDN

For peer review only

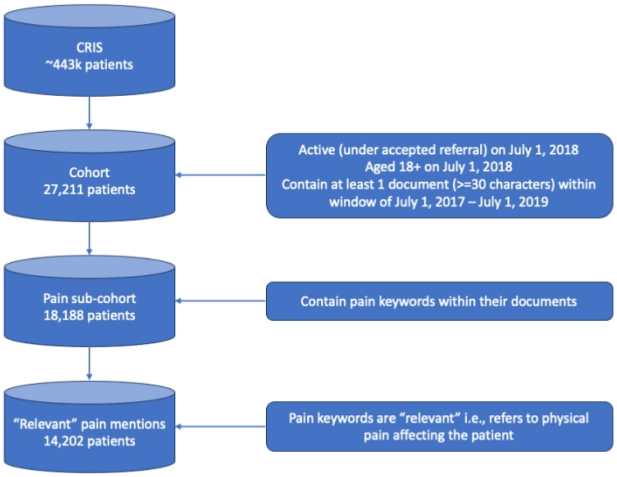

Figure 1. Data Extraction

339x191mm (102 x 102 DPI)

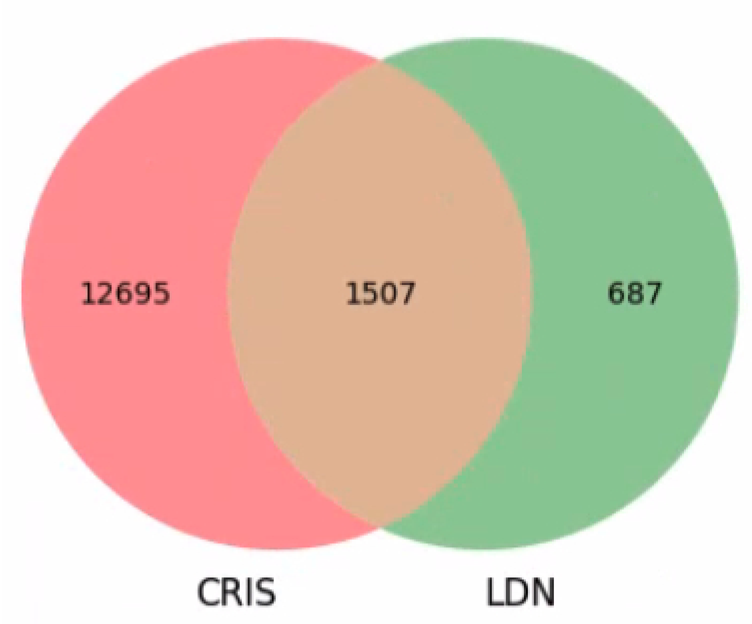

Figure 2. Overlap of recorded pain in CRIS and LDN

132x116mm (72 x 72 DPI)

Reporting checklist for cross sectional study.

Based on the STROBE cross sectional guidelines.

Instructions to authors

Complete this checklist by entering the page numbers from your manuscript where readers will find each of the items listed below.

Your article may not currently address all the items on the checklist. Please modify your text to include the missing information. If you are certain that an item does not apply, please write "n/a" and provide a short explanation.

Upload your completed checklist as an extra file when you submit to a journal.

In your methods section, say that you used the STROBE cross sectionalreporting guidelines, and cite them as:

von Elm E, Altman DG, Egger M, Pocock SJ, Gotzsche PC, Vandenbroucke JP. The Strengthening the Reporting of Observational Studies in Epidemiology (STROBE) Statement: guidelines for reporting observational studies.

For peer review only

		Reporting Item	Page Number
**Title and abstract**			
Title	#1a	Indicate the study's design with a commonly used term in the title or the abstract	1
Abstract	#1b	Provide in the abstract an informative and balanced summary of what was done and what was found	1
**Introduction**			
Background / rationale	#2	Explain the scientific background and rationale for the investigation being reported	2
Objectives	#3	State specific objectives, including any prespecified hypotheses	4
**Methods**			
Study design	#4	Present key elements of study design early in the paper	4
Setting	#5	Describe the setting, locations, and relevant dates, including periods of	4

		recruitment, exposure, follow-up, and data collection	
Eligibility criteria	#6a	Give the eligibility criteria, and the sources and methods of selection of participants.	5
	#7	Clearly define all outcomes, exposures, predictors, potential confounders, and effect modifiers. Give diagnostic criteria, if applicable	5
Data sources / measurement	#8	For each variable of interest give sources of data and details of methods of assessment (measurement). Describe comparability of assessment methods if there is more than one group. Give information separately for for exposed and unexposed groups if applicable.	5-7
Bias	#9	Describe any efforts to address potential sources of bias	14
Study size	#10	Explain how the study size was arrived at	5
Quantitative variables	#11	Explain how quantitative variables were handled in the analyses. If applicable, describe which groupings were chosen, and why	6
Statistical methods	#12a	Describe all statistical methods, including those used to control for confounding	8
Statistical methods	#12b	Describe any methods used to examine subgroups and interactions	8
Statistical methods	#12c	Explain how missing data were addressed	6
Statistical methods	#12d	If applicable, describe analytical methods taking account of sampling strategy	n/a
Statistical methods	#12e	Describe any sensitivity analyses	n/a
**Results**			
Participants	#13a	Report numbers of individuals at each stage of study—eg numbers potentially eligible, examined for eligibility, confirmed eligible, included in the study, completing follow-up, and analysed. Give information separately for for exposed and unexposed groups if applicable.	8
Participants	#13b	Give reasons for non-participation at each stage	8
Participants	#13c	Consider use of a flow diagram	8

For peer review only

Descriptive data	#14a	Give characteristics of study participants (eg demographic, clinical, social) and information on exposures and potential confounders. Give information separately for exposed and unexposed groups if applicable.	8-9
Descriptive data	#14b	Indicate number of participants with missing data for each variable of interest	9
Outcome data	#15	Report numbers of outcome events or summary measures. Give information separately for exposed and unexposed groups if applicable.	9-10
Main results	#16a	Give unadjusted estimates and, if applicable, confounder-adjusted estimates and their precision (eg, 95% confidence interval). Make clear which confounders were adjusted for and why they were included	10-11
Main results	#16b	Report category boundaries when continuous variables were categorized	11
Main results	#16c	If relevant, consider translating estimates of relative risk into absolute risk for a meaningful time period	n/a
Other analyses	#17	Report other analyses done—e.g., analyses of subgroups and interactions, and sensitivity analyses	12-13
**Discussion**			
Key results	#18	Summarise key results with reference to study objectives	13
Limitations	#19	Discuss limitations of the study, taking into account sources of potential bias or imprecision. Discuss both direction and magnitude of any potential bias.	13-15
Interpretation	#20	Give a cautious overall interpretation considering objectives, limitations, multiplicity of analyses, results from similar studies, and other relevant evidence.	13-15
Generalisability	#21	Discuss the generalisability (external validity) of the study results	13-15
**Other Information**			
Funding	#22	Give the source of funding and the role of the funders for the present study and, if applicable, for the original study on which the present article is based	16

The STROBE checklist is distributed under the terms of the Creative Commons Attribution License CC-BY. This checklist was completed on 24. August 2023 using https://www.goodreports.org/, a tool made by the EQUATOR Network in collaboration with Penelope.ai

For peer review only

The RECORD statement – checklist of items, extended from the STROBE statement, that should be reported in observational studies using routinely collected health data.

	Item No.	STROBE items	Location in manuscript where items are reported	RECORD items	Location in manuscript where items are reported
**Title and abstract**					
	1	(a) Indicate the study's design with a commonly used term in the title or the abstract (b) Provide in the abstract an informative and balanced summary of what was done and what was found	(a) Title<br>(b) Abstract	RECORD 1.1: The type of data used should be specified in the title or abstract. When possible, the name of the databases used should be included.<br><br>RECORD 1.2: If applicable, the geographic region and timeframe within which the study took place should be reported in the title or abstract.<br><br>RECORD 1.3: If linkage between databases was conducted for the study, this should be clearly stated in the title or abstract.	Abstract
**Introduction**					
Background rationale	2	Explain the scientific background and rationale for the investigation being reported	Introduction - Background Rationale		
Objectives	3	State specific objectives, including any prespecified hypotheses	Introduction - Objectives		
**Methods**					
Study Design	4	Present key elements of study design early in the paper	Methods		
Setting	5	Describe the setting, locations, and relevant dates, including periods of recruitment, exposure, follow-up, and data collection	Methods - Setting and Variables		

For peer review only

Participants	6	*(a) Cohort study* - Give the eligibility criteria, and the sources and methods of selection of participants. Describe methods of follow-up<br>*Case-control study* - Give the eligibility criteria, and the sources and methods of case ascertainment and control selection. Give the rationale for the choice of cases and controls<br>*Cross-sectional study* - Give the eligibility criteria, and the sources and methods of selection of participants<br><br>*(b) Cohort study* - For matched studies, give matching criteria and number of exposed and unexposed<br>*Case-control study* - For matched studies, give matching criteria and the number of controls per case	Methods - Participants	RECORD 6.1: The methods of study population selection (such as codes or algorithms used to identify subjects) should be listed in detail. If this is not possible, an explanation should be provided.<br><br>RECORD 6.2: Any validation studies of the codes or algorithms used to select the population should be referenced. If validation was conducted for this study and not published elsewhere, detailed methods and results should be provided.<br><br>RECORD 6.3: If the study involved linkage of databases, consider use of a flow diagram or other graphical display to demonstrate the data linkage process, including the number of individuals with linked data at each stage.	
Variables	7	Clearly define all outcomes, exposures, predictors, potential confounders, and effect modifiers. Give diagnostic criteria, if applicable.	Methods - Variables	RECORD 7.1: A complete list of codes and algorithms used to classify exposures, outcomes, confounders, and effect modifiers should be provided. If these cannot be reported, an explanation should be provided.	
Data sources/ measurement	8	For each variable of interest, give sources of data and details of methods of assessment (measurement).<br>Describe comparability of assessment methods if there is more than one group	Methods - Variables		

For peer review only

Bias	9	Describe any efforts to address potential sources of bias	Methods - Variables		
Study size	10	Explain how the study size was arrived at	Methods - Participants		
Quantitative variables	11	Explain how quantitative variables were handled in the analyses. If applicable, describe which groupings were chosen, and why	Methods - Variables		
Statistical methods	12	(a) Describe all statistical methods, including those used to control for confounding<br>(b) Describe any methods used to examine subgroups and interactions<br>(c) Explain how missing data were addressed<br>(d) *Cohort study* - If applicable, explain how loss to follow-up was addressed<br>*Case-control study* - If applicable, explain how matching of cases and controls was addressed<br>*Cross-sectional study* - If applicable, describe analytical methods taking account of sampling strategy<br>(e) Describe any sensitivity analyses	Methods - Setting and Descriptive Statistics		
Data access and cleaning methods		..		RECORD 12.1: Authors should describe the extent to which the investigators had access to the database population used to create the study population.	Methods - Setting - Ethical Approval

For peer review only

				RECORD 12.2: Authors should provide information on the data cleaning methods used in the study.	Methods - Variables
Linkage		..		RECORD 12.3: State whether the study included person-level, institutional-level, or other data linkage across two or more databases. The methods of linkage and methods of linkage quality evaluation should be provided.	Methods - Setting
**Results**					
Participants	13	(a) Report the numbers of individuals at each stage of the study (*e.g.*, numbers potentially eligible, examined for eligibility, confirmed eligible, included in the study, completing follow-up, and analysed)<br>(b) Give reasons for non-participation at each stage.<br>(c) Consider use of a flow diagram	Results	RECORD 13.1: Describe in detail the selection of the persons included in the study (*i.e.*, study population selection) including filtering based on data quality, data availability and linkage. The selection of included persons can be described in the text and/or by means of the study flow diagram.	Methods
Descriptive data	14	(a) Give characteristics of study participants (*e.g.*, demographic, clinical, social) and information on exposures and potential confounders<br>(b) Indicate the number of participants with missing data for each variable of interest<br>(c) *Cohort study* - summarise follow-up time (*e.g.*, average and total amount)	Results		
Outcome data	15	*Cohort study* - Report numbers of outcome events or summary measures over time<br>*Case-control study* - Report numbers in each exposure	Results		

For peer review only

		category, or summary measures of exposure<br>*Cross-sectional study* - Report numbers of outcome events or summary measures			
Main results	16	(a) Give unadjusted estimates and, if applicable, confounder-adjusted estimates and their precision (e.g., 95% confidence interval). Make clear which confounders were adjusted for and why they were included<br>(b) Report category boundaries when continuous variables were categorized<br>(c) If relevant, consider translating estimates of relative risk into absolute risk for a meaningful time period	Results		
Other analyses	17	Report other analyses done—e.g., analyses of subgroups and interactions, and sensitivity analyses	Results		
**Discussion**					
Key results	18	Summarise key results with reference to study objectives	Discussion		
Limitations	19	Discuss limitations of the study, taking into account sources of potential bias or imprecision. Discuss both direction and magnitude of any potential bias	Discussion	RECORD 19.1: Discuss the implications of using data that were not created or collected to answer the specific research question(s). Include discussion of misclassification bias, unmeasured confounding, missing data, and changing eligibility over time, as they pertain to the study being reported.	Discussion and Conclusion
Interpretation	20	Give a cautious overall interpretation of results considering objectives,			

For peer review only

		limitations, multiplicity of analyses, results from similar studies, and other relevant evidence	Discussion		
Generalisability	21	Discuss the generalisability (external validity) of the study results	Discussion		
**Other Information**					
Funding	22	Give the source of funding and the role of the funders for the present study and, if applicable, for the original study on which the present article is based	Acknolwledgements and disclosure of interests		
Accessibility of protocol, raw data, and programming code		..	Data Availability Statement	RECORD 22.1: Authors should provide information on how to access any supplemental information such as the study protocol, raw data, or programming code.	Data Availability Statement

*Reference: Benchimol EI, Smeeth L, Guttmann A, Harron K, Moher D, Petersen I, Sørensen HT, von Elm E, Langan SM, the RECORD Working Committee. The REporting of studies Conducted using Observational Routinely-collected health Data (RECORD) Statement. *PLoS Medicine* 2015; in press.

*Checklist is protected under Creative Commons Attribution (CC BY) license.

For peer review only
